# Mindfulness-Based Psychoeducation App to Improve the Well-Being of Parents and Caregivers of Children With Autism: Development and Usability Study

**DOI:** 10.2196/84224

**Published:** 2026-06-04

**Authors:** Karen KY Ma, Sandra SM Chan, Elisabeth WY Wong, Olivia Choi, Flora YM Mo, Caroline KS Shea, Carol SW Ho, Patrick WL Leung, Suzanne HW So, Susan M Bögels, Florrie FY Ng, Steven WH Chau, Kelly YC Lai, Gabriel PC Fung, Oscar WH Wong

**Affiliations:** 1 Department of Psychiatry, Faculty of Medicine, The Chinese University of Hong Kong, Hong Kong, China (Hong Kong); 2 MRC Epidemiology Unit, School of Clinical Medicine, University of Cambridge, Box 285 Institute of Metabolic Science, Cambridge Biomedical Campus, Cambridge, CB2 0QQ, United Kingdom, 44 1223 330315; 3 Department of Psychiatry, Alice Ho Miu Ling Nethersole Hospital, Hong Kong, China (Hong Kong); 4 Department of Psychology, Faculty of Social Science, The Chinese University of Hong Kong, Hong Kong, China (Hong Kong); 5 Child Development and Education, University of Amsterdam, Amsterdam, The Netherlands; 6 Department of Educational Psychology, Faculty of Education, The Chinese University of Hong Kong, Hong Kong, China (Hong Kong); 7 Department of Systems Engineering and Engineering Management, Faculty of Engineering, The Chinese University of Hong Kong, Hong Kong, China (Hong Kong)

**Keywords:** mindfulness, digital intervention, autism, caregiver stress, mental wellbeing

## Abstract

**Background:**

Autism spectrum disorder (ASD) not only affects a person’s social communication and behaviors, but also has an impact on their parents, who encounter different challenges during caregiving. Interventions developed for parents of children with ASD often focus on improving child outcomes and seldom consider the well-being of parents and families. Interventions leveraging mindfulness-based approaches have been developed to support parents of children with ASD, but the costs, inflexibility, and scarcity of resources may limit their accessibility. App-based interventions can be an accessible, scalable, and economical way of providing interventions at a primary health care level.

**Objective:**

The aim of this study was to develop an evidence-based digital intervention that complements existing, overloaded psychiatric services, to provide mindfulness-based psychoeducation for parents of children with ASD to improve their mental well-being.

**Methods:**

The app development process follows the systematic approach of intervention mapping. Needs assessment was first conducted through semistructured qualitative interviews with health care professionals. Performance and change objectives were specified; theory-based and practical application methods were selected, followed by the design of the curriculum for a structured intervention. A pilot waitlist randomized controlled trial was conducted to evaluate the feasibility, acceptability, and preliminary efficacy of the app with parents of children with ASD recruited from a tertiary child psychiatric service in Hong Kong.

**Results:**

The resulting intervention, the TRIP app, is a 6-week structured intervention consisting of 6 sessions per week (each session lasting 15‐20 minutes), covering topics on ASD parenting skills and mindfulness practices. The six weekly themes include (1) cultivating curiosity in parenting, (2) mindfulness of the breath and body, (3) management of core and associated features of ASD, (4) managing conflicts and setting boundaries, (5) perspective taking, and (6) cultivating self-compassion. The curriculum was designed to target the determinants of parental stress, including parents’ knowledge, skills, emotions, and attitudes. App content and features were designed to incorporate behavioral change techniques, social cognitive theory, and elaboration likelihood model, to enhance efficacy and promote long-term usage. The app was found to be feasible and acceptable in the pilot randomized controlled trial (n=40), with greater long-term usage among parents of children on the waiting list who were yet to receive diagnostic assessment and clinical management, when compared with parents of children who have already been receiving clinical care.

**Conclusions:**

The TRIP app was developed based on knowledge and expertise across psychiatry, public health, behavioral science, and implementation science. It caters to the unmet needs for improving caregiver well-being in the holistic care model for families of children with ASD. The clinical efficacy of the TRIP app is yet to be evaluated through clinical trials.

## Introduction

Autism spectrum disorder (ASD) is a neurodevelopmental disorder that can affect individuals throughout their lifetime, as persistent deficits and behavioral challenges can lead to functional impairments [1], and may also have a vast impact on their parents and families. Current interventions for ASD are primarily focused on improving adaptive functioning and ameliorating maladaptive behaviors [2], often comprising a combination of behavioral interventions, social communication training, and sensorimotor therapy. The delivery of these interventions is often brief and infrequent, limited by both the capacity of health service providers and the costs of the interventions. As a result, parents and caregivers inevitably take on a role in delivering interventions. Parent training programs (eg, the widely known Triple P Positive Parenting Program [3] and the Incredible Years Parent Training program [4]) were adapted to equip parents of children with ASD with the required skills for independent implementation of interventions with minimal or no support from therapists. While parent training for ASD is effective in improving child development [3,5,6], most of these trainings focused on improving children’s behaviors and functioning, with a lack of emphasis on alleviating parental stress or enhancing their mental well-being and quality of life. As parents tend to shoulder a great deal of stress with limited professional support, taking care of parents’ own mental well-being is essential when supporting parents in implementing interventions for their children [7].

In terms of the approaches used in interventions developed for supporting parents, mindfulness-based interventions (MBIs) were found to have positive effects in reducing parental stress and depressive symptoms and improving psychological well-being in parents of children with ASD [8,9]. Interventions tailored to integrate mindfulness into parenting for ASD have been developed, such as the Mindful Parenting program, which has the following aims: (1) to reduce parental stress, negative bias, and reactivity; (2) to break the cycle of intergenerational continuity of maladaptive parenting; (3) to promote self-nourishing attention and self-compassion; (4) to improve marital relationships, functioning, co-parenting; and (5) to reduce interparental conflicts [10]. The Mindful Parenting program was later extended into the MYmind program for ASD, delivering parallel, concomitant mindfulness training for adolescents with ASD and their parents, which was found to improve parenting style and competence, and increase quality of life for both adolescents and their parents [11].

Although MBIs have promising outcomes, the fixed 8-week structure of the courses may not fit into parents’ busy schedules and could be costly, especially for families of lower socioeconomic status. App-based interventions can overcome these barriers and provide an accessible, flexible, and affordable platform for education and lifestyle changes. Furthermore, digital platforms can enable easier and quicker access to intervention content. Prompts through navigation, notification, and gamification can also encourage access to content compared with traditional web-based interventions [12]. A recent meta-analysis demonstrated that app-based mindfulness interventions were efficacious in reducing perceived stress and improving symptoms of depression and anxiety across diverse samples, including the general adult population and clinical populations suffering from major medical illnesses [13]. Some randomized controlled trials (RCTs) even showed comparable effects between app-based and face-to-face interventions [14]. Recent studies also found that app-based mindfulness interventions were effective for parents, as studies reported improvements in mindful parenting in the general population [15] and increased positive parent-child interactions and reduced parenting stress among parents of children with ASD [16]. Therefore, app-based interventions can be an accessible and economical way of providing a feasible alternative to in-person interventions for improving parental well-being.

While the market is not deprived of generic smartphone apps for improving mental health or mindfulness, attrition and non-adherence are common in unguided app-based interventions [17,18]. As app-based interventions were found to be more efficacious when complementing current care and being customized to users’ goals and needs [19], tailored content is a crucial element in maintaining motivation for continual use. To date, the only app-based intervention developed for parents of children with ASD is the Smartautism app, a mobile app developed in France that uses ecological momentary assessment (EMA) to provide feedback for parents of children with ASD (aged 3‐16 y) to adapt parents’ educational behavior between medical appointments [20,21], and no app-based interventions have been developed for delivering MBIs tailored for parents of children with ASD. Therefore, considering the limited resources and inadequate provision in public health care for ASD, the aim of this study was to develop an app for mindfulness-based psychoeducation for parents and caregivers of children with ASD, and to evaluate the feasibility, acceptability, and preliminary efficacy of the app. Specifically, while the prevalence of ASD in Hong Kong is comparable to that of Western countries [22], clinical services for ASD in Hong Kong have seen a rapid increase in attendance by 162% over the past decade, with a 3-fold increase in average waiting time from 23 weeks in 2012‐2013 to 80 weeks in 2019‐2020 [23]. As such, we would like to explore whether the preliminary efficacy of the app is different for parents of children who were still on the waiting list to be seen by a psychiatrist for diagnostic assessments and clinical management, and hence are receiving lower levels of care and support when compared with parents of children who have already been receiving clinical care.

## Methods

### Overview

**Figure 1.**
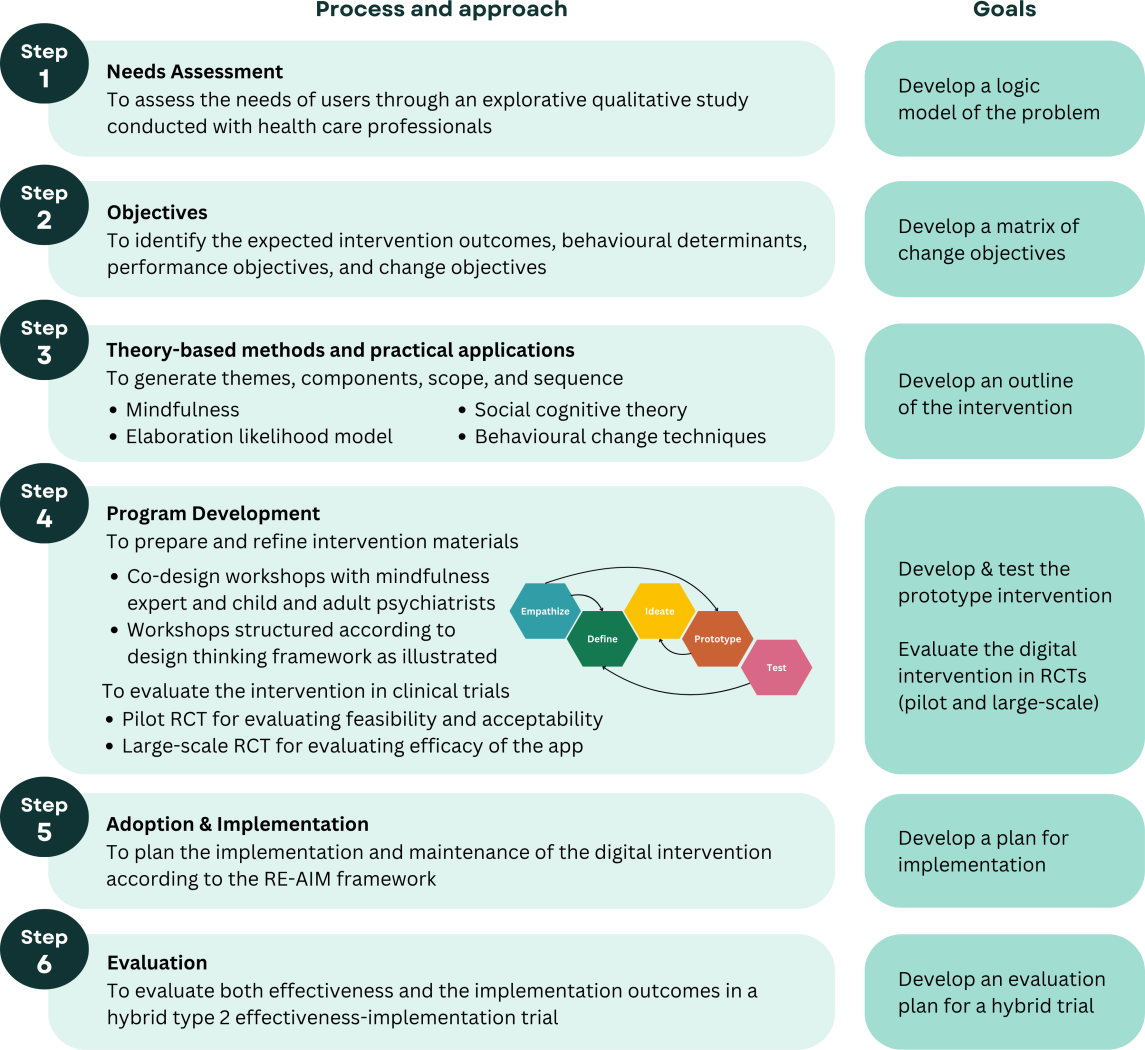
Systematic development of the TRIP app using intervention mapping. RCT: randomized controlled trial; RE-AIM: Reach, Effectiveness, Adoption, Implementation, and Maintenance.

The app development process follows a systematic, evidence-informed approach according to intervention mapping (IM) [24,25], a protocol for intervention development that fulfills the UK Medical Research Council framework for developing complex health care interventions [26]. IM is an iterative, 6-step method that is well-established and widely used (Figure 1). This article focuses on reporting the results of steps 1‐4 of the IM process, including the development and usability testing of the app. Steps 5‐6, which involve intervention implementation and evaluation, are planned but will be reported separately when completed. This report follows the Consolidated Standards of Reporting Trials of Electronic and Mobile Health Applications and Online Telehealth (CONSORT-EHEALTH) checklist (V.1.6.1).

### Ethical Considerations

Ethical approval was obtained from The Joint Clinical Research Ethics Committee established by The Chinese University of Hong Kong and New Territories East Cluster (qualitative study in step 1: Ref 2021.591; pilot RCT in step 4: 2022.586). Informed consent was obtained from all participants according to the Declaration of Helsinki. Data were anonymized and securely stored to ensure participant confidentiality. Upon the completion of the study, health care professionals in step 1 did not receive any financial compensation, whereas parents in step 4 received vouchers valued at HKD $100 (approximately US $12.50).

### Step 1: Needs Assessment

The needs of parents and caregivers of children with ASD were assessed through an explorative qualitative study conducted with health care professionals to understand the underlying sources of challenges faced by ASD caregivers and recommendations to support parents in overcoming these challenges, complementing the lived experiences of parents and families that are already well-documented in the literature. Health care professionals providing tertiary specialist clinical services for ASD were purposively sampled via referral by professional acquaintances, and the sample size was guided by the principle of information power [27]. Semistructured interviews were conducted according to an interview guide developed with input from psychiatrists based on clinical experiences (Multimedia Appendix 1). Topics included management of ASD symptoms, impact of ASD on family dynamics, experiences of stigma around ASD, and responses toward the diagnosis. Open-ended questions and supplementary probing were used to allow any explorative, spontaneous, and in-depth discussions that may emerge during the conversation [28]. Interviews were first transcribed verbatim and checked for accuracy. Qualitative data then underwent inductive coding [29,30]. Codes were then mapped onto an adapted social ecological model (SEM), which considers multiple levels of influence on health and well-being, including individual, interpersonal, institutional, community, and societal levels [31]. Codes were then compiled to create a logic model based on the PRECEDE model (ie, Predisposing, Reinforcing, and Enabling Constructs in Educational Diagnosis and Evaluation), which provides a comprehensive framework for assessing the different determinants of health through an educational diagnosis before developing and implementing interventions for health promotion [32]. The PRECEDE model comprises 4 phases, beginning with a social assessment, followed by an epidemiological assessment, a behavioral and environmental assessment, and an educational and ecological assessment. Detailed methods and findings from the subsequent thematic analysis of this qualitative study were published separately [33].

### Step 2: Matrices of Change Objectives

Based on the results from step 1, intervention outcomes and relevant, modifiable behavioral determinants were identified. This was used for specifying the performance objectives, which are specific actions required to achieve the intervention outcome. A matrix of change objectives was then created, which specifically identifies behaviors or thought processes that needed to be changed in order to achieve the target outcomes.

### Step 3: Theory-Based Methods and Practical Applications

Theory-based methods and practical applications were selected to achieve behavior change in parents and caregivers of children with ASD. As there is considerable evidence in the existing literature for the effectiveness of MBIs in supporting ASD caregivers, mindfulness was selected as the basis for content development. The elaboration likelihood model (ELM) [34], which posits a dual process theory of attitude change through central and peripheral processing requiring varying levels of cognitive efforts, was selected as the basis for curriculum development. The social cognitive theory (SCT) [35], which suggests that people can learn to change, achieve, and maintain goal-directed behaviors over time through different constructs, including behavioral capability, outcome expectations, observational learning, self-efficacy, and reinforcements, was selected as the basis for the design of app content and features. Behavior change techniques (BCTs) based on an extensive taxonomy of 93 distinct techniques across 16 different group categories by Michie and colleagues [36] were mapped onto different SCT constructs for practical application to target specific change objectives.

### Step 4: Program Development

#### App Development and Evaluation Process

The digital intervention was developed by a collaborative team of researchers, psychiatrists, mindfulness experts, software engineers, graphic designers, and text editors. The app development process involves the parallel, iterative development of app curriculum and content, content production and refinement, and software design and development. App curriculum and content were developed through 10 co-design sessions (each lasting around an hour) with an experienced mindfulness teacher (EWYW), a child psychiatrist (OWHW), an adult psychiatrist (SSMC), a doctoral researcher with a psychology background (KKYM), and our project manager (OC). Co-design sessions were structured according to the design thinking framework [37], with a focus on iterative cycles of define, ideate, and prototyping for the design of the app curriculum and content. Content production and refinement was done in collaboration with a text editor for editing and proofreading, a mindfulness expert for audio recording of mindfulness practices and voice-over narrations for animations, and a graphic designer for illustrations and animations. Software design and development was actualized by software engineers and graphic designers, respectively. The prototype of the app underwent alpha and beta testing with health care professionals and parents before it was officially launched. A prospectively registered pilot waitlist-RCT [38] was then conducted to evaluate the feasibility, acceptability, and preliminary efficacy of the app. This was followed by an ongoing large-scale RCT for evaluating the efficacy of the app (n=700) [39], which will be reported separately when the trial is completed.

#### Participant Recruitment

Participants were 40 parents or caregivers of children with ASD (aged ≤12 y) diagnosed by clinicians according to the *DSM-5* (*Diagnostic and Statistical Manual of Mental Disorders* [Fifth Edition]) criteria [40]. Only parents who can understand spoken instructions in Cantonese and texts written in traditional Chinese were eligible. Parents who were not primary caregivers, or those currently receiving psychological interventions or undergoing mindfulness training, were excluded. Participants were recruited from a university-affiliated tertiary hospital providing the sole public child and adolescent psychiatric service in a region of >1.2 million population in Hong Kong. In the clinic, parents were recruited from either the triage service (where the child had gone through initial triage assessment by nurses but were still on the waiting list to be seen by a psychiatrist for diagnostic assessments and clinical management), or the psychiatrist-led clinic (where ASD diagnosis was already made and the child was receiving active treatment and training from the hospital), therefore representing 2 different groups of parents whose children were receiving different levels of care and support.

Potential participants were approached individually in a short call via videoconferencing, where they were given information about the study and had their questions addressed. Eligibility of interested participants was screened through a brief screener survey. Written informed consent was obtained from all eligible participants who agreed to participate. Participants were randomly allocated to either the immediate intervention group or the waitlist-control group, using the Qualtrics randomizer with a 1:1 allocation ratio through stratified block randomization (block sizes of 4, stratified according to the level of support received [waiting list vs clinical care] and child age [≤6 y, 7-9 y, and 10‐12 y]). Participants in both groups received the intervention: the intervention group received the intervention immediately after allocation, whereas the control group received the intervention after 6 weeks of treatment as usual.

#### Sociodemographic and Clinical Characteristics

Sociodemographic information (including parents’ sex, age, and educational level, and child’s sex and age) and clinical characteristics were obtained at baseline. Child clinical characteristics were considered by parent-reported measures, including the Social Responsiveness Scale, second edition (SRS-2) for ASD core symptoms [41], as well as the Anxiety Scale for Children-ASD questionnaire [42] and the externalizing and attention problems subscales of the Child Behavior Checklist (CBCL-Ext and CBCL-Attn) [43] for common co-occurring psychopathology. Age-appropriate versions of SRS-2 and CBCL were used for preschool and school-aged children, respectively.

#### Outcome Measures

The primary outcomes of the pilot study were feasibility and acceptability of the app. Feasibility was measured by app usage during the intervention period, including usage patterns (day of week and time of day), user engagement (number of usage days, number of logins, and time spent per day), program participation (number of sessions completed and curriculum completion rate), and retention rates in terms of current use (participants who used the app during a given week) and last use (the last time the app was used by participants). Acceptability was assessed immediately post intervention by the 10-item System Usability Scale (SUS-10) [44], assessing the usability of products and apps, and the 20-item User Version of the Mobile Application Rating Scale (uMARS) [45], measuring engagement, functionality, aesthetics, and information quality of health apps.

The secondary outcome was preliminary efficacy, including parental mental health, parenting capabilities, and levels of mindfulness measured at baseline, preintervention, postintervention, and at 2-month follow-up. The selection of outcome measures was informed by the social and epidemiological assessment in step 1. Parental mental health was assessed by the 7-item General Anxiety Disorder scale (GAD-7) [46] and the 9-item Patient Health Questionnaire (PHQ-9) [47], and parenting stress was measured by the 36-item Parenting Stress Index-Short Form (PSI-SF) [48]. Parenting capabilities were considered in terms of parenting competence and satisfaction, which were measured by the 17-item Parenting Sense of Competence scale (PSOC) [49], the 6-item parenting efficacy subscale of the Parenting Self-Agency measure (PSA-PE) [50], and the 7-item warmth subscale of the Parenting Styles and Dimensions Questionnaire (PSDQ-W) [51]. Levels of mindfulness were assessed by the 15-item Mindful Attention Awareness Scale (MAAS) [52,53] and the 31-item Interpersonal Mindfulness in Parenting questionnaire (IM-P) [54,55].

#### Statistical Analysis

Analysis of covariance (adjusting for baseline values) was used to investigate the intervention effect and outcome changes from preintervention. Analyses were conducted according to the intention-to-treat principle, with last observation carried forward for any missing data. Analyses were stratified by the source of recruitment (triage service or psychiatrist-led clinic), as this represents different levels of support received by parents of children with ASD (waiting list vs clinical care). Intervention effect on all efficacy outcomes was analyzed separately with the same procedures, with outcome scores as the dependent variable, randomization group as a factor, and baseline values of the outcome and child clinical characteristics (SRS-2 and CBCL-Ext) as covariates (based on previous findings from the literature [56,57]). A similar procedure was used to compare changes in outcomes from preintervention to postintervention and 2-month follow-up, with assessment timepoint as a factor, and adjusting for preintervention value of the outcome, child clinical characteristics at baseline (SRS-2 and CBCL-Ext), and randomization group, to account for any potential confounding effects in intervention participation as a result of delayed intervention [58]. All analyses were performed in jamovi (version 2.6.2; The jamovi project) [59].

## Results

### Step 1: Needs Assessment

We conducted 10 interviews, including 7 individual interviews and 3 group interviews, with 16 health care professionals working in different roles, including psychiatrists (n=2), psychiatric nurses (n=9), clinical psychologists (n=2), an occupational therapist (n=1), a hospital-based teacher (n=1), and a social worker (n=1). We identified 4 key areas at the individual level of the SEM where parents faced challenges during caregiving, namely knowledge, skills, emotions, and attitudes. The key caregiving skills needed were broadly categorized into skills for managing specific child behaviors, skills during daily interactions with children, and skills for self-care. Moreover, health care professionals also shared their perspectives on wider contextual and environmental factors within the SEM that pose challenges for parents when caring for children with ASD. These include interpersonal factors such as inconsistent parenting and autism in the family, institutional factors such as the transition from kindergarten to primary school and gaps in clinical service provision, community factors such as support systems and cultural influences, and societal factors related to stigma surrounding ASD. Detailed codes and examples are provided in Multimedia Appendix 2. Detailed findings from the subsequent thematic analysis of this qualitative study were published separately [33].

**Figure 2.**
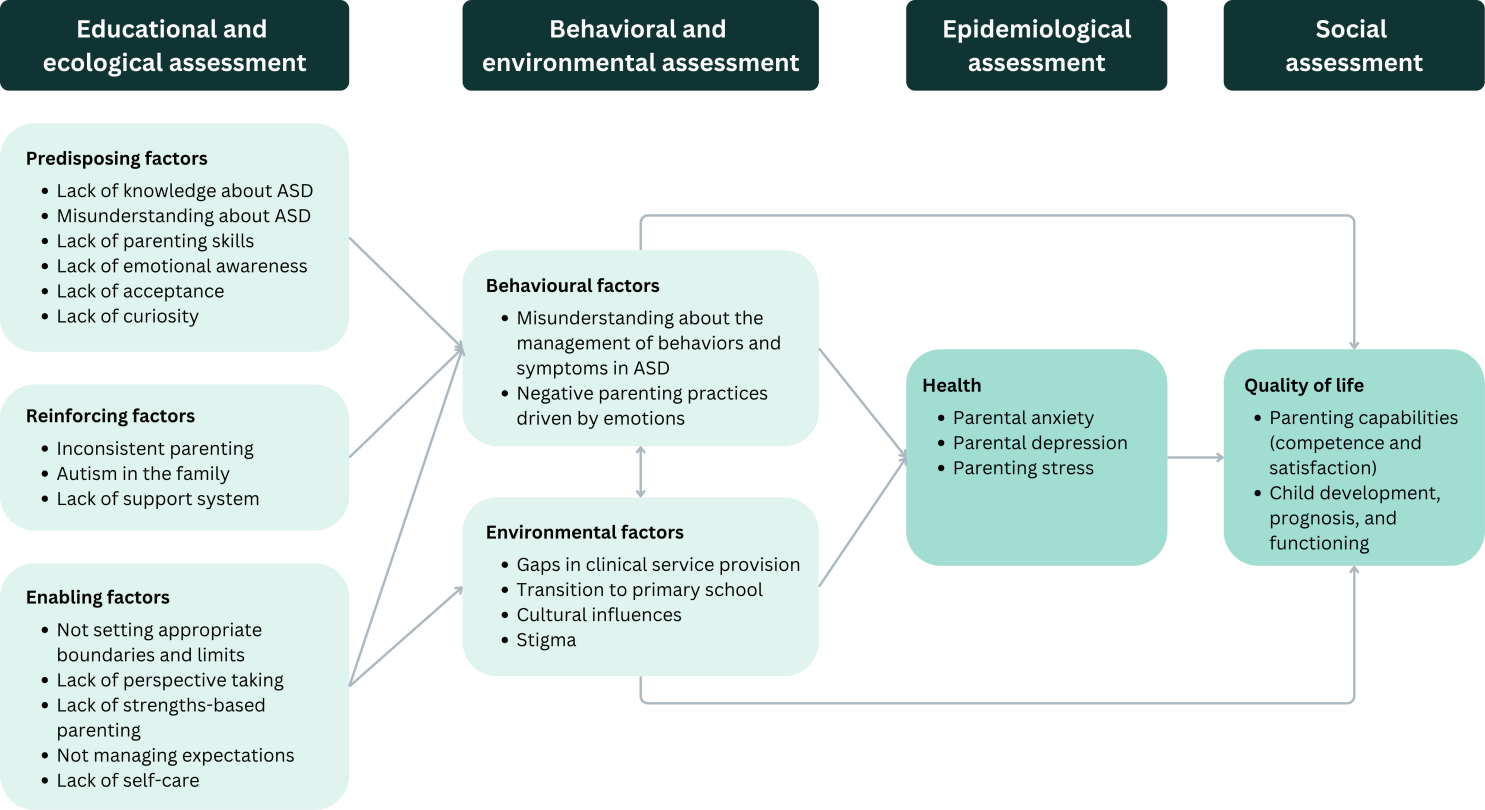
Logic model for caregiver challenges faced by parents of children with autism spectrum disorder. ASD: autism spectrum disorder.

Figure 2 shows a logic model for caregiver challenges faced by parents of children with ASD. Social assessment identified aspects of quality of life impacted by caregiving for ASD, including parenting competence and satisfaction, and children’s development, prognosis, and functioning. Epidemiological assessment suggested that caregiver challenges affect parents’ health through parenting stress, anxiety, and depression. Parallel analyses of behavioral and environmental factors revealed that misunderstanding about the management of behaviors and symptoms and negative parenting practices driven by emotions were the main challenges faced by parents, which were influenced by various environmental factors, including service gap, school transitions, cultural influences, and stigma. Educational and ecological diagnosis identified the main needs of parents in terms of predisposing factors related to parents’ knowledge, skills, attitudes, and emotions, reinforcing factors in parents’ social environment, and enabling factors related to varying levels of relational skills for parenting.

### Step 2: Matrices of Change Objectives

Based on the findings from the needs assessment in step 1, the overall intervention outcome was to reduce parental stress, anxiety, and depression, whereas the behavioral outcome was to improve parents’ management of children’s behaviors and symptoms. The specific performance objectives for the behavioral outcome were to (1) improve parents’ understanding of behaviors and symptoms, and (2) improve parents’ emotional regulation. A matrix of change objectives for parents of children with ASD based on the specific performance objectives is presented in Table 1.

**Table 1.** Matrix of change objectives for parents of children with ASD^a^.

Performance	Change objectives categorized by determinants^b^			
objectives	Knowledge	Skills	Emotions	Attitudes
To improve parents’ understanding of behaviors and symptoms	Improve knowledge about ASD around sensory issues and academic abilities (1.1.1) Address misunderstandings about ASD related to social communication, repetitive behaviors, emotional expression, and comorbidity (1.1.2) Learn about specific skills for toilet training, communication skills training, and organizing play (1.1.3)	Use effective instructions and commands when parenting (1.2.1) Avoid misconstrued behavioral reinforcement and encourage parents to make use of children’s stereotyped interests (1.2.2) Adopt an investigative mindset during parent-child interactions and take children’s perspective to understand their underlying intentions and motivations for their behaviors (1.2.3)	Avoid emotionally-charged parenting, especially when trying to understand children’s behaviors (1.3.1)	Increase curiosity in children’s behaviors and characteristics (1.4.1)
To improve parents’ emotional regulation	Understand the negative effects of unhelpful parenting practices on the parent-child relationship and children’s development (2.1.1) Understand how inconsistent parenting can be confusing and unhelpful for children (2.1.2)	Avoid emotionally-charged parenting and other negative parenting practices and adopt strengths-based parenting (2.2.1) Manage own expectations about children’s progress and development (2.2.2) Balance between showing love and setting limits (2.2.3) Reserve time for self-care through cultivating parents’ own interests and hobbies (2.2.4)	Improve emotional awareness (2.3.1) Increase self-compassion (2.3.2)	Acknowledge the presence of ASD diagnosis (2.4.1) Increase acceptance toward ASD (2.4.2)

a ASD: autism spectrum disorder.

b Codes (eg, 1.1.1, 1.1.2, and so on) are with reference to Multimedia Appendix 3.

### Step 3: Theory-Based Methods and Practical Applications

#### Mindfulness

As mindfulness is underpinned by key mechanisms of decentering and self-compassion, mindfulness practices for training attention control, decentering, and self-compassion, alongside practices that were further contextualized to parenting for ASD, were included in the app. This is important, as decentering can shift one’s cognitive perspective to help reduce rumination [60,61], whereas self-compassion can increase positive affect and emotions for improving psychological well-being [62-64]. According to the warp and weft framework [65] that outlines the core essential elements and adaptable variable elements in MBIs, we preserved as many of the core essential elements in the curriculum and the content of the app. As direct inquiry is not possible in a self-taught format, the experiential, inquiry-based learning process was adapted by including debriefs that were intended to help parents reflect on their experiences and develop insight and understanding following the completion of guided mindfulness practices. Home practice was supported through convenient options for revisiting mindfulness practices through the intervention.

#### ELM

As ELM emphasizes how information processing may be done through the central or peripheral route depending on one’s motivation and can lead to varying degrees of attitude changes, we developed intervention content on mindfulness and parenting for ASD that varied in depth and complexity (and therefore required different levels of cognitive efforts) to accommodate parents with different levels of motivation and cognitive abilities. For mindfulness content, gratitude practices, self-compassion practices, and attentional control training require less cognitive effort, whereas decentering practices, cognitive exercises, and relational skills require more cognitive effort. For psychoeducation content on parenting for ASD, knowledge transfer components require less cognitive effort, whereas skills transfer components, especially those explaining fundamental principles in psychological and behavioral science, require more cognitive effort.

#### SCT

**Figure 3.**
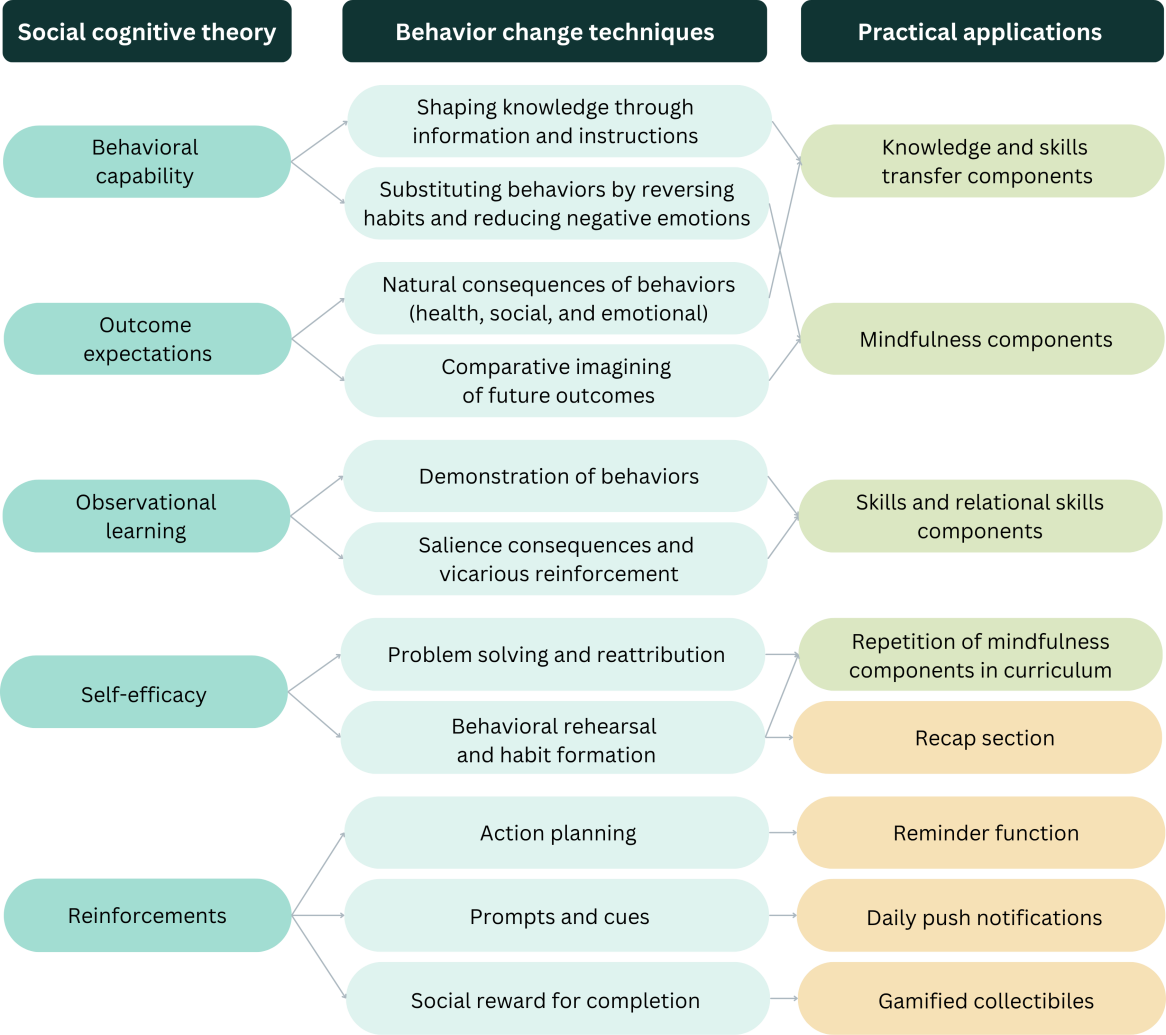
Social cognitive theory constructs mapped onto behavior change techniques for practical application.

Figure 3 illustrates how BCTs were selected and applied to different SCT constructs (with details of how they map to specific change objectives provided in Multimedia Appendix 3). Intervention content for mindfulness and parenting for ASD was designed to target behavioral capability, outcome expectations, and observational learning. App features were designed to facilitate self-efficacy and reinforcements. For instance, we dedicated a section of the app to hold the entire collection of learning components to allow convenient revisiting and repeated practice. We also included a reminder function to help users set aside a specific time for completing the intervention, at which daily push notifications were sent to prompt users to log in and complete a session.

### Step 4: Program Development

**Figure 4.**
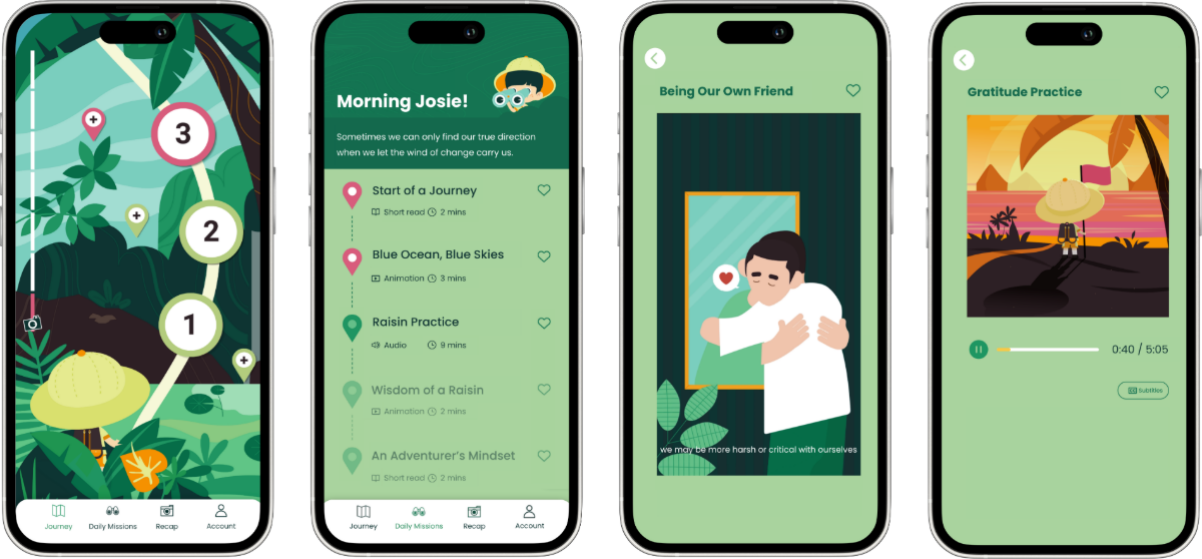
Main screens of the TRIP app translated into English.

**Figure 5.**
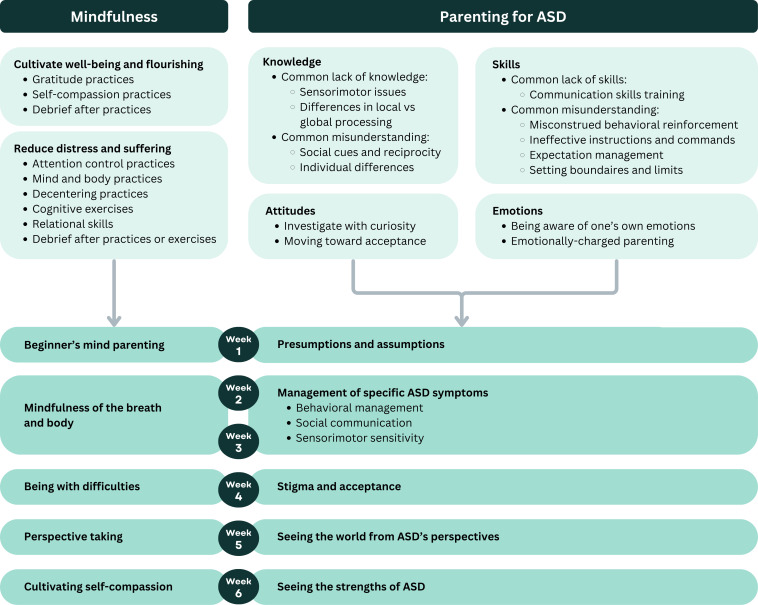
Outline of the themes and components of the TRIP app. ASD: autism spectrum disorder.

As “investigating with curiosity” was a recurring theme across interviews with health care professionals in step 1, the app was named TRIP and was designed as a journey for parents to learn more about themselves and their children through the cultivation of mindfulness and curiosity. The TRIP app is a 6-week structured intervention, with each week featuring a specific theme on mindfulness and parenting for ASD. Each week consists of 6 sessions, each session lasting 15‐20 minutes; core and optional sessions are delivered on alternate days, with core sessions covering new content in greater detail, whereas optional sessions reinforce key messages of the week through self-directed learning. Within each session, learning components were delivered in different formats, including narrated animations (total duration=31 min, 7%), audio-guided meditation practices (total duration=307 min, 69%), and short articles (total duration=107 min, 24%). Each learning component focuses on either mindfulness practices, exercises, debriefing, and relational skills, or knowledge, skills, emotions, or attitudes in parenting for ASD, corresponding to the selected determinants of ASD caregiver challenges as identified in step 2. The app is currently available on App Store and Google Play in Chinese, with texts written in traditional Chinese characters and audio spoken in Cantonese, a popular Chinese dialect widely spoken in Hong Kong. Figure 4 illustrates the main screens of the TRIP app, and Figure 5 illustrates an outline of the themes and the intervention.

In the pilot RCT, 117 participants were screened for eligibility. A total of 77 parents were excluded, as they did not meet the eligibility criteria (n=5), declined to participate (n=24), or did not respond to the screener survey (n=48). In total, 40 parents (90% female; 20 parents of children on the waiting list and 20 parents of children already receiving clinical care) were enrolled and randomized, with 20 allocated to the intervention group, and 20 allocated to the control group (2 parents dropped out during follow-up, both were parents of children already receiving clinical care, and one allocated to the intervention group and the other to the control group). Most participants were female (n=36, 90%), while their children with ASD were mostly male (n=30, 75%) with a mean age of 5.18 (SD 2.46) years. There were no significant differences in participants’ characteristics between the intervention and control groups (*P* values>.05). Multimedia Appendix 4 shows the CONSORT-EHEALTH flow diagram of the study, participants’ characteristics at baseline, and detailed results for feasibility, acceptability, and preliminary efficacy outcomes.

**Figure 6.**
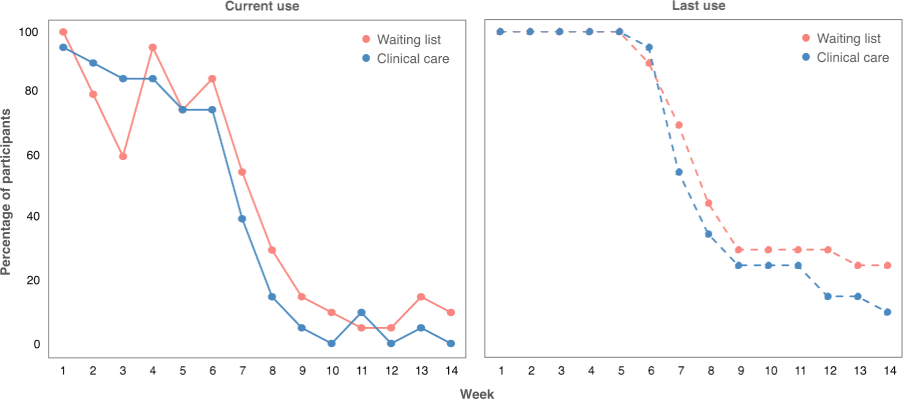
Week-by-week representation of participants’ usage pattern during the active intervention period (weeks 1‐6) and 2-month follow-up period (weeks 7‐14).

For primary outcomes of feasibility and acceptability, the 6-week app-based structured intervention was both feasible and acceptable for supporting parents of children with ASD. Overall, the usage pattern is similar for parents receiving different levels of support during the active intervention period, whereas parents of children on the waiting list had a greater level of continued usage in the 2-month follow-up period than parents of children already receiving clinical care (Figure 6). All participants used the app during weeks 1‐4, 92.5% (37/40) were still using the app at week 6, and 17.5% (7/40) continued using the app after the active intervention period. On average, participants completed 78.7% (65.3/83 unique components, SD 11.51) of the curriculum and 93.9% (16.9/18, SD 2.48) of the core sessions in the active intervention period, although the completion rate for optional sessions was rather low at 13.3% (2.4/18, SD 2.60). The overall usability of the system received a mean SUS score of 79.1 (SD 9.76) out of 100, reflecting “good” usability on the scale. The overall app quality based on the mean uMARS score was 4.09 (SD 0.41) out of 5. The app is highly rated for information (mean 4.52, SD 0.43), functionality (mean 4.43, SD 0.42), and aesthetics (mean 4.12, SD 0.55), whereas the rating for engagement is slightly lower (mean 3.29, SD 0.56).

**Figure 7.**
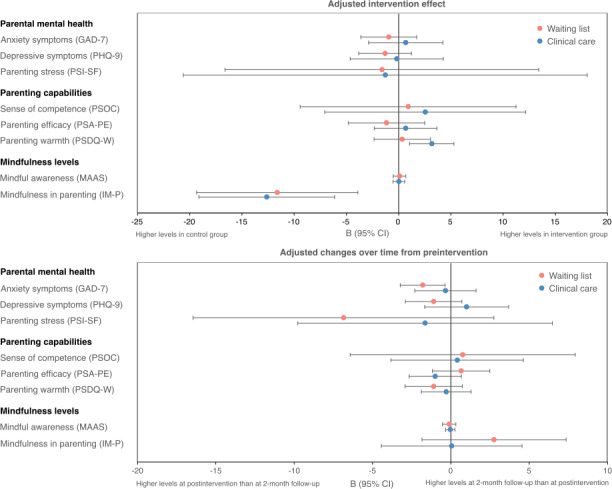
Intervention effect and outcome changes over time from preintervention to postintervention and 2-month follow-up for efficacy outcomes, stratified by the levels of support received (waiting list vs clinical care). GAD-7: 7-item General Anxiety Disorder scale; PHQ-9: 9-item Patient Health Questionnaire; PSI-SF: 36-item Parenting Stress Index-Short Form; PSOC: 17-item Parenting Sense of Competence scale; PSA-PE: 6-item parenting efficacy subscale of the Parenting Self-Agency measure; PSDQ-W: 7-item warmth subscale of the Parenting Styles and Dimensions Questionnaire; MAAS: 15-item Mindful Attention Awareness Scale; IM-P: 31-item Interpersonal Mindfulness in Parenting questionnaire.

For the secondary outcome of preliminary efficacy, the intervention effect and outcome changes from preintervention to postintervention and 2-month follow-up are illustrated in Figure 7 (refer to Multimedia Appendix 4 for full results). There was no evidence of intervention effects on parents’ mental health (GAD-7, PHQ-9, and PSI-SF), parenting capabilities (PSOC, PSA-PE, and PSDQ-W), and mindfulness levels (MAAS and IM-P) at 6-week postbaseline for parents receiving different levels of support, except for increased parenting warmth (PSDQ-W) among parents of children already receiving clinical care (B=3.17, 95% CI 1.04-5.30). Moreover, improvements in parenting warmth (PSDQ-W) were more favorable among parents of children who have already been receiving clinical care, whereas reductions in parental anxiety (GAD-7) and depression (PHQ-9) were more favorable among parents of children on the waiting list who were yet to receive diagnostic assessment and clinical management. Unexpectedly, there was a greater increase in the levels of mindfulness in parenting (IM-P) in the control group (parents of children on the waiting list: B=−11.62, 95% CI −19.34 to −3.90; parents of children already receiving clinical care: B=−12.63, 95% CI −19.13 to −6.13), although the CI was wide and, therefore, suggests uncertainties in the precision of these estimates. Comparing the outcome changes from preintervention to postintervention and 2-month follow-up, there were no notable continual improvements in most outcomes, except for a further decrease in parental anxiety (GAD-7) among parents of children on the waiting list (B=−1.80, 95% CI −3.23 to −0.37). Nonetheless, trends in outcome changes over time showed favorable reductions in parental depressive symptoms (PHQ-9) and improvements in parenting efficacy (PSA-PE) at 2-month follow-up for parents of children on the waiting list when compared with postintervention, whereas there were few changes in these outcomes from preintervention to 2-month follow-up for parents of children already receiving clinical care.

## Discussion

### Principal Findings

In this study, we used a systematic, evidence-informed approach according to IM to develop an accessible and scalable digital intervention to support parents and caregivers of children with ASD. To our knowledge, this is the first project of its kind in the field of autism, creating a family-centered intervention to be implemented in primary care that complements existing clinical services and helps bridge service gaps in addressing unmet needs in the overloaded public specialist service system. This project is particularly intended to support families of children who are still on the waiting list to be seen by a psychiatrist for diagnostic assessments and clinical management. The digital format of the intervention also has great potential in reaching and providing an alternative avenue of care for those who are reluctant to seek conventional treatment due to concerns over privacy or mental health stigma. As the potential impact of improved parental mental well-being extends beyond parents themselves, this project can contribute to the effective management of elevated levels of parental stress in families of children with ASD and is potentially beneficial to both family functioning and child development in the long-term.

Through the IM process, the TRIP app was developed based on evidence-based research, incorporating existing findings from the literature and novel results from the explorative qualitative study with health care professionals conducted for needs assessment. Compared with MBIs developed for face-to-face or online synchronous delivery, the format of a digital platform posed constraints on the app content that can be covered, which may be limited in terms of depth and intensity to minimize the risk of harm or adverse events beyond the common, expected discomfort associated with meditation. For example, when exploring the theme of rupture and repair, instead of the more emotionally intense imaginary exercises in the Mindful Parenting program that may be potentially triggering, we created case studies of lower emotional intensity for observational learning to bring out the same learning points. In a similar vein, while research has emphasized the importance of partner relationships in parenting, it was not practical to address sensitive or highly individualized issues within an app-based format. As such, we have avoided the topic of partner relationships in the app, due to concerns about potential misuse between parents, including the possibility of parents undermining one another, or exacerbating conflicts between parents or separated coparents. Finally, the absence of a group learning environment, which is typical of MBIs, may limit the emergence of themes that often arise through collaborative exploration, such as the recognition of shared experiences of distress as part of our common humanity. This is because the sense of mutual understanding that arises in group settings can be particularly helpful for processing difficult emotions and realizations. Therefore, it would be interesting to implement app-based interventions within a group context, such as through peer support groups or similar networks in community settings, which is planned for later steps.

### Feasibility and Acceptability of the App

Results from the pilot study suggest that the TRIP app is a feasible and acceptable way of delivering self-guided mindfulness-based psychoeducation for parents of children with ASD. All participants were actively engaged in the first 5 weeks of the active intervention period, with a low attrition rate of 7.5% at the end of the 6-week intervention, compared with a weighted meta-analytic attrition rate of 24.7% from 78 RCTs of mindfulness apps [66]. During the follow-up period, more than half of the participants revisited some learning components, and just under a third remained active users, with higher engagement among parents of children on the waiting list than parents of children already receiving clinical care. This is somewhat comparable to the engagement in mindfulness-based meditation apps in individuals awaiting psychological services [67]. Likewise, the completion rates of core sessions were substantially higher than those of optional sessions, likely because participants viewed optional sessions as nonessential, akin to the follow-up period. These observed usage patterns may be due to intrinsic factors related to the intervention structure, as the fixed course of core sessions for participants to progress through may have encouraged engagement and compliance, whereas optional sessions positioned outside of the core course scheduled with similar frequencies may have induced fatigue. Similarly, the learning components being available for revisiting in the follow-up period may have created decision-paralysis, thereby contributing to attrition from regular use. However, it is also possible that participants did not have the time to complete optional sessions during the week or did not feel the need to revisit any of the learning components in the follow-up period; therefore, spacing out optional sessions in the intervention period or releasing new learning components regularly in the follow-up period may increase engagement. On the other hand, extrinsic factors such as the study context, including support from research staff during the intervention period, may have acted as both a reminder and a source of accountability, thereby accounting for the increased app usage in the intervention period, as well as the decline in app usage in the follow-up period. This is in line with findings from a rapid scoping review suggesting that improved engagement with digital MBIs may be associated with additional support and encouragement from therapists, researchers, significant others, or other participants [68]. As such, this has been incorporated in the implementation plan, with staff across different settings included as implementers.

### Preliminary Efficacy of the App

While we found that the app is feasible and acceptable for parents, there is little evidence for the preliminary efficacy of the app beyond the significant increase in parenting warmth among parents of children already receiving clinical care. An obvious explanation is that the pilot RCT is underpowered for detecting any intervention effects as a result of its small sample size. That said, trends observed in the data suggest that the app may potentially have different effects depending on the level of support children were receiving, as the app may be effective in reducing anxiety and depression in parents of children on the waiting list and increasing parenting efficacy in parents of children already receiving clinical care (in addition to a significant increase in parenting warmth). Moreover, the intervention effects may be delayed in parents of children on the waiting list, which was evident for further decrease in parental anxiety from postintervention to 2-month follow-up and indicated by similar trends observed for reductions in parental depression and improvements in parenting efficacy. This might be due to parents of children on the waiting list receiving minimal clinical support and, therefore, taking more time to understand and apply the knowledge and skills learned in the app to their everyday parenting. However, this means that any intervention effects beyond the active intervention period may not be detected, as comparisons with controls were only made during the active intervention period. Trends for outcome changes from postintervention to 2-month follow-up also suggest that the app may bring greater changes to parents of children already receiving clinical care during the intervention period, as reductions in parental depression and improvements in parenting efficacy reverted to preintervention levels at 2-month follow-up. It will be interesting to further investigate this through conducting subgroup analyses with an adequately powered sample in the ongoing large-scale RCT.

Moreover, it may also be possible that the TRIP app may have effects that were not assessed by the outcome measures used in this study. We included measures of parental mental health and parenting capabilities in terms of parenting competence and efficacy, which were selected based on results of the explorative qualitative study. While outcomes selection in this study was in line with established frameworks for integrating parents and family into research and practice [7], measures of parenting behaviors, family interactions, or family quality of life were not included in this study and could be incorporated in the future to provide a more comprehensive evaluation of intervention efficacy and effectiveness. Also, while validated measurement tools were developed for measuring knowledge about ASD in the general population [69] and knowledge of parenting strategies [70], no instruments have been adapted to measure knowledge or skills in caregiving for ASD beyond autism-specific parenting self-efficacy [71]. As such, we did not examine whether there were increases in knowledge or skills for caregiving for ASD, as it would have been difficult to operationalize these changes in a valid and reliable manner in the absence of appropriate psychometric tools.

With regard to the unexpected effects of the app attenuating the natural increase in the levels of mindfulness in parenting, a plausible explanation for this draws on the conscious-competence learning model for the process of experiential learning [72]. As this outcome was measured by self-report, it is possible that participants were in the unconscious-incompetence stage at preintervention, in which they may not be aware of how little they know, and thus have unknowingly inflated their estimations of their levels of mindfulness in parenting. Subsequently, through the TRIP app, they may have progressed to the conscious-incompetence stage as their understanding of mindful parenting improves, and they began to recognize that they might not be as mindful as they had previously thought, thereby explaining the stark decrease in levels of mindfulness in parenting in the intervention group from preintervention to postintervention. In other words, parents may have become more aware of their nonmindful parenting as a result of using the TRIP app. Subsequently, through increased practice and application of skills acquired from the app, parents may have progressed to the conscious-competence stage, which may explain the slight increase in levels of mindfulness in parenting from postintervention to 2-month follow-up. This highlights the need to consider how to best operationalize changes in skills that can be learned over time, especially in light of the individual differences in learning and awareness.

### Strengths and Limitations

This study offers several strengths. The approach for app development is systematic, evidence-based, and the use of a clinician-led, co-design approach enables the resulting digital intervention to be family-centered and complement existing clinical services. Moreover, the randomized waitlist-controlled design for the pilot study has the merits of conferring high internal validity and maintaining the gold standard of evidence without withholding the intervention from participants in the control group, offering a more ethical way of intervention testing. Apart from taking into account several important confounding factors (eg, child age and clinical characteristics), careful consideration of the role of the source of recruitment (triage service vs psychiatrist-led clinic) allowed us to gain valuable insights into how families receiving different levels of support may interact with and use the app differently, potentially contributing to differential intervention effects. The inclusion of server-side documented data also allowed us to understand how the app was used, as assessed by objective measures.

While this project has many strengths, there are also various limitations that need to be acknowledged. First, the high nonresponse rates may introduce sampling bias and potentially limit the generalizability of our findings. Moreover, it was possible that parents of children who were on the waiting list may have attended their first appointment with a psychiatrist for diagnostic assessment and clinical management during the intervention period. While psychiatric care is typically a long-term process that may take more time than the 6-week intervention period to show observable effects, any emerging improvements could have confounded the outcomes measured in the pilot study. However, the sample size in the pilot study was too small to adequately explore whether results varied by changes in the level of support received during the study period (eg, transitioning from waiting list to clinical care) or whether results vary by ASD severity. Furthermore, due to time and resource constraints, it was not possible to adopt a mixed methods approach for the pilot study. It would have been interesting to obtain qualitative data from participants to explore the factors influencing their compliance, which may inform how the app can be better implemented in the future to promote continual use beyond the 6-week structured intervention. In a similar vein, it was not practical to extend the waiting period in the waitlist control group beyond the length of the structured intervention, which meant that intervention effects were only compared during the 6-week intervention period and did not allow for any delayed effects to be compared.

### Direction for Future Research

To address the potential biases and confounders that may arise in the pilot study, the ongoing large-scale RCT should compare whether response rates differ across key subgroups within the sample, explore whether results varied by ASD severity and changes in the level of support received from clinical services, and adjust for these factors accordingly. To overcome the limitation of the lack of direct evidence on parents’ lived experience and qualitative user feedback in the pilot study, factors associated with enhanced user experiences and long-term usage will be explored at the end of the ongoing RCT through qualitative interviews with parents and observations of naturalistic usage patterns beyond the active intervention period. Quantitative server-side documented data on app usage in the 6 months following the active intervention period will be analyzed to explore factors associated with long-term usage. Individual qualitative interviews (each lasting 30‐60 min) will be conducted with parents to explore their user experience of the app, their subjective parenting experience, and their relationship with their children during the intervention period. The interviews will also seek feedback on how the content and delivery can be improved, and identify factors that may promote or hinder compliance with the intervention program. Results from the qualitative interviews will be used to refine the TRIP app before it is translated into English to complete the program development (step 4).

For adoption and implementation (step 5), we plan to implement the app according to a stepwise approach, commencing with implementation in clinical settings for families who are underserved by the public tertiary psychiatric services, followed by wider dissemination in the community through nonprofit organizations and local community centers, in order to be inclusive of those who may identify as neurodiverse but are unbeknownst to clinical services. A detailed implementation plan was devised as guided by the Reach, Effectiveness, Adoption, Implementation, and Maintenance (RE-AIM) framework [73] (Multimedia Appendix 5).

For program evaluation (step 6), a hybrid type 2 effectiveness-implementation trial will be conducted to evaluate both effectiveness and implementation outcomes simultaneously [74]. Implementers at different sites will be recruited and randomized to either the early implementation group or the delayed implementation group. Those who are allocated to the early implementation group will receive training and support for the implementation of the app immediately after randomization, whereas those allocated to the delayed implementation group will receive the implementation intervention once they have recruited the target number of parents of children with ASD. Implementation outcomes assessing feasibility, acceptability, fidelity, and sustainability will be measured at the end of the implementation period for the early implementation group, or immediately after randomization for the delayed implementation group, as well as at 3-month and 6-month follow-up after the first measurement. Effectiveness outcomes for mental health and quality of life outcomes for parents of children with ASD will be assessed at preintervention, postintervention, and at 3-month follow-up.

Apart from the planned implementation and evaluation, future studies can focus on investigating the mechanisms of change brought by the app by including measures of intermediate outcomes, such as attention and self-compassion. Future research can also include objective psychophysiological measures of depression and mindfulness alongside self-report measures by incorporating EMA, which was found to be more sensitive than paper-and-pencil measures [75].

### Conclusions

In this study, we created an evidence-based digital intervention for parents of children with autism with an interdisciplinary approach at the heart of psychiatry, public health, and behavioral and implementation science. We demonstrated how we can incorporate behavior change theories and techniques while leveraging the experiences of clinicians for the coproduction of a feasible and acceptable digital intervention that complements existing clinical services in addressing pressing clinical needs. The TRIP app has great potential to be an economical, first-tier intervention in the stepped-care model for the holistic management of autism, by bridging service gaps through supporting parents of children awaiting diagnostic assessment and clinical care, and by improving resource allocation within existing clinical services through freeing up capacity to focus on complex cases that require more specialized, individualized, and intensive input.

## Supplementary material

10.2196/84224 Multimedia Appendix 1 Qualitative study topic guide for semistructured interviews.

10.2196/84224 Multimedia Appendix 2 Qualitative study codes and examples mapped to an adapted socioecological model.

10.2196/84224 Multimedia Appendix 3 Practical application of social cognitive theory and behavior change techniques to achieve change objectives.

10.2196/84224 Multimedia Appendix 4 Pilot waitlist-randomized controlled trial recruitment flow, participants' baseline characteristics, and full results tables.

10.2196/84224 Multimedia Appendix 5 Implementation plan for different settings.

## Abbreviations

ASD: autism spectrum disorder
BCT: behavior change techniques
CBCL: Child Behavior Checklist
CBCL-Attn: Attention Problems Scale of the Child Behavior Checklist
CBCL-Ext: Externalizing Problem Scale of the Child Behavior Checklist
CONSORT-EHEALTH: Consolidated Standards of Reporting Trials of Electronic and Mobile Health Applications and Online Telehealth
*DSM-5*: *Diagnostic and Statistical Manual of Mental Disorders* (Fifth Edition)
ELM: elaboration likelihood model
EMA: ecological momentary assessment
GAD: 7-item Generalized Anxiety Disorder Scale
IM: intervention mapping
IM-P: 31-item Interpersonal Mindfulness in Parenting Questionnaire
MAAS: 15-item Mindful Attention Awareness Scale
MBI: mindfulness-based intervention
PHQ: 9-item Patient Health Questionnaire
PRECEDE: Predisposing, Reinforcing, and Enabling Constructs in Educational Diagnosis and Evaluation
PSA-PE: 6-item Parenting Efficacy Subcale of the Parenting Self-Agency Measure
PSDQ-W: 7-item Warmth Subscale of the Parenting Styles and Dimensions Questionnaire
PSI-SF: 36-item Parenting Stress Index-Short Form
PSOC: 17-item Parenting Sense of Competence Scale
RCT: randomized controlled trial
RE-AIM: Reach, Effectiveness, Adoption, Implementation, and Maintenance
SCT: social cognitive theory
SEM: social ecological model
SRS-2: Social Responsiveness Scale, second edition
SUS: 10-item System Usability Scale
uMARS: 20-item User Version of the Mobile Application Rating Scale

## References

[1] Steinhausen HC, Mohr Jensen C, Lauritsen MB (2016). A systematic review and meta-analysis of the long-term overall outcome of autism spectrum disorders in adolescence and adulthood. Acta Psychiatr Scand.

[2] Mukherjee SB (2017). Autism spectrum disorders - diagnosis and management. Indian J Pediatr.

[3] Whittingham K, Sofronoff K, Sheffield J, Sanders MR (2009). Stepping Stones Triple P: an RCT of a parenting program with parents of a child diagnosed with an autism spectrum disorder. J Abnorm Child Psychol.

[4] Webster-Stratton C, Dababnah S, Olson E (2018). The Incredible Years® Group-Based Parenting Program for Young Children with Autism Spectrum Disorder.

[5] McGilloway S, Mhaille GN, Bywater T (2012). A parenting intervention for childhood behavioral problems: a randomized controlled trial in disadvantaged community-based settings. J Consult Clin Psychol.

[6] Webster-Stratton C (1998). Preventing conduct problems in Head Start children: strengthening parenting competencies. J Consult Clin Psychol.

[7] Wainer AL, Hepburn S, McMahon Griffith E (2017). Remembering parents in parent-mediated early intervention: an approach to examining impact on parents and families. Autism.

[8] Cachia RL, Anderson A, Moore DW (2016). Mindfulness, stress and well-being in parents of children with autism spectrum disorder: a systematic review. J Child Fam Stud.

[9] Li SN, Chien WT, Lam SKK, Chen ZY, Ma X (2024). Effectiveness of parent-focused interventions for improving the mental health of parents and their children with autism spectrum disorder: a systematic review and meta-analysis. Res Autism Spectr Disord.

[10] Bögels SM, Lehtonen A, Restifo K (2010). Mindful parenting in mental health care. Mindfulness (N Y).

[11] de Bruin EI, Blom R, Smit FM, van Steensel FJ, Bögels SM (2015). MYmind: mindfulness training for youngsters with autism spectrum disorders and their parents. Autism.

[12] Morrison LG, Geraghty AWA, Lloyd S (2018). Comparing usage of a web and app stress management intervention: an observational study. Internet Interv.

[13] Gál É, Ștefan S, Cristea IA (2021). The efficacy of mindfulness meditation apps in enhancing users’ well-being and mental health related outcomes: a meta-analysis of randomized controlled trials. J Affect Disord.

[14] Compen F, Bisseling E, Schellekens M (2018). Face-to-face and internet-based mindfulness-based cognitive therapy compared with treatment as usual in reducing psychological distress in patients with cancer: a multicenter randomized controlled trial. JCO.

[15] Warner LW (2022). Using a Mindfulness Smartphone App to Reduce Parenting Stress and Increase Mindful Parenting.

[16] Phan J (2022). Effects of a Brief Mobile Mindfulness Application on Mindful Parenting, Noncompliance of Children with and without Autism Spectrum Disorder, Perceived Parenting Stress, and ParentChild Interactions.

[17] Kelders SM, Kok RN, Ossebaard HC, Van Gemert-Pijnen J (2012). Persuasive system design does matter: a systematic review of adherence to web-based interventions. J Med Internet Res.

[18] Ludden GDS, van Rompay TJL, Kelders SM, van Gemert-Pijnen J (2015). How to increase reach and adherence of web-based interventions: a design research viewpoint. J Med Internet Res.

[19] Torous J, Jän Myrick K, Rauseo-Ricupero N, Firth J (2020). Digital mental health and COVID-19: using technology today to accelerate the curve on access and quality tomorrow. JMIR Ment Health.

[20] Bonnot O, Bonneau D, Doudard A, Duverger P (2016). Rationale and protocol for using a smartphone application to study autism spectrum disorders: SMARTAUTISM. BMJ Open.

[21] Bonnot O, Adrien V, Venelle V, Bonneau D, Gollier-Briant F, Mouchabac S (2021). Mobile app for parental empowerment for caregivers of children with autism spectrum disorders: prospective open trial. JMIR Ment Health.

[22] Wong OW, Sm Chan S, Chau SW (2025). Autism epidemiology in Hong Kong children and youths aged 6-17: implications on autism screening and sex differences in the community. Autism.

[23] Lam C (2021). Mental health services for young people. Legislative Council of the Hong Kong Special Administration Region of the People’s Republic of China.

[24] Bartholomew Eldredge LK, Markham CM, Ruiter RAC, Fernández ME, Kok G, Parcel GS Planning Health Promotion Programs: An Intervention Mapping Approach.

[25] Fernandez ME, Ruiter RAC, Markham CM, Kok G (2019). Intervention mapping: theory- and evidence-based health promotion program planning: perspective and examples. Front Public Health.

[26] Craig P, Dieppe P, Macintyre S (2008). Developing and evaluating complex interventions: the new Medical Research Council guidance. BMJ.

[27] Malterud K, Siersma VD, Guassora AD (2016). Sample size in qualitative interview studies: guided by information power. Qual Health Res.

[28] Ryan F, Coughlan M, Cronin P (2009). Interviewing in qualitative research: the one-to-one interview. Int J Ther Rehabil.

[29] Braun V, Clarke V (2006). Using thematic analysis in psychology. Qual Res Psychol.

[30] Braun V, Clarke V (2019). Reflecting on reflexive thematic analysis. Qualitative Research in Sport, Exercise and Health.

[31] McLeroy KR, Bibeau D, Steckler A, Glanz K (1988). An ecological perspective on health promotion programs. Health Educ Q.

[32] Green LW, Kreuter MW (2005). Health Program Planning: An Educational and Ecological Approach.

[33] Ma KKY, Burn AM, Wong OWH, Choi O, Chan SSM (2026). Healthcare professionals’ perspectives on the challenges faced by parents of children with autism and recommendations to address them: a qualitative study in Hong Kong. BMJ Paeds Open.

[34] Petty RE, Cacioppo JT (1986). The elaboration likelihood model of persuasion. Adv Exp Soc Psychol.

[35] Bandura A (1986). Social Foundations of Thought and Action: A Social Cognitive Theory.

[36] Michie S, Richardson M, Johnston M (2013). The Behavior Change Technique Taxonomy (v1) of 93 hierarchically clustered techniques: building an international consensus for the reporting of behavior change interventions. Ann Behav Med.

[37] Plattner H An introduction to design thinking - process guide. Stanford University.

[38] Pilot testing of a mobile phone app-based intervention for parents of children with autism spectrum disorder (ASD). ClinicalTrials.gov.

[39] RCT of a mobile phone app-based intervention for parents of children with autism spectrum disorder (ASD). ClinicalTrials.gov.

[40] Regier DA, Kuhl EA, Kupfer DJ (2013). The DSM-5: classification and criteria changes. World Psychiatry.

[41] Gau SSF, Liu LT, Wu YY, Chiu YN, Tsai WC (2013). Psychometric properties of the Chinese version of the Social Responsiveness Scale. Res Autism Spectr Disord.

[42] Rodgers J, Wigham S, McConachie H, Freeston M, Honey E, Parr JR (2016). Development of the anxiety scale for children with autism spectrum disorder (ASC-ASD). Autism Res.

[43] Leung PWL, Kwong SL, Tang CP (2006). Test–retest reliability and criterion validity of the Chinese version of CBCL, TRF, and YSR. Child Psychology Psychiatry.

[44] Brooke J, Jordan P, Thomas T, Weerdmeester B (1996). Usability Evaluation in Industry.

[45] Stoyanov SR, Hides L, Kavanagh DJ, Wilson H (2016). Development and validation of the user version of the Mobile Application Rating Scale (uMARS). JMIR Mhealth Uhealth.

[46] Spitzer RL, Kroenke K, Williams JBW, Löwe B (2006). A brief measure for assessing generalized anxiety disorder: the GAD-7. Arch Intern Med.

[47] Kroenke K, Spitzer RL, Williams JBW (2001). The PHQ-9. J Gen Intern Med.

[48] Haskett ME, Ahern LS, Ward CS, Allaire JC (2006). Factor structure and validity of the parenting stress index-short form. J Clin Child Adolesc Psychol.

[49] Ohan JL, Leung DW, Johnston C (2000). The Parenting Sense of Competence Scale: evidence of a stable factor structure and validity. Canadian Journal of Behavioural Science / Revue canadienne des sciences du comportement.

[50] Dumka LE, Stoerzinger HD, Jackson KM, Roosa MW (1996). Examination of the cross-cultural and cross-language equivalence of the parenting self-agency measure. Fam Relat.

[51] Robinson CC, Mandleco B, Olsen SF, Hart CH (1995). Authoritative, authoritarian, and permissive parenting practices: development of a new measure. Psychol Rep.

[52] MacKillop J, Anderson EJ (2007). Further psychometric validation of the Mindful Attention Awareness Scale (MAAS). J Psychopathol Behav Assess.

[53] Deng YQ, Li S, Tang YY, Zhu LH, Ryan R, Brown K (2012). Psychometric properties of the Chinese Translation of the Mindful Attention Awareness Scale (MAAS). Mindfulness (N Y).

[54] Duncan LG (2007). Assessment of Mindful Parenting among Parents of Early Adolescents: Development and Validation of the Interpersonal Mindfulness in Parenting Scale.

[55] Lo HHM, Yeung JWK, Duncan LG (2018). Validating of the Interpersonal Mindfulness in Parenting Scale in Hong Kong Chinese. Mindfulness (N Y).

[56] McStay RL, Trembath D, Dissanayake C (2014). Stress and family quality of life in parents of children with autism spectrum disorder: parent gender and the double ABCX model. J Autism Dev Disord.

[57] Pozo P, Sarriá E, Brioso A (2014). Family quality of life and psychological well-being in parents of children with autism spectrum disorders: a double ABCX model. J Intellect Disabil Res.

[58] Gunnarsson KU, McCambridge J, Bendtsen M (2023). Reactions to being allocated to a waiting list control group in a digital alcohol intervention trial. Patient Educ Couns.

[59] (2024). Jamovi (version 25) [computer software]. The jamovi project (2025).

[60] Hayes-Skelton S, Graham J (2013). Decentering as a common link among mindfulness, cognitive reappraisal, and social anxiety. Behav Cogn Psychother.

[61] Teasdale JD, Moore RG, Hayhurst H, Pope M, Williams S, Segal ZV (2002). Metacognitive awareness and prevention of relapse in depression: empirical evidence. J Consult Clin Psychol.

[62] Fredrickson BL (2004). The broaden-and-build theory of positive emotions. Philos Trans R Soc Lond B Biol Sci.

[63] Neff KD, Rude SS, Kirkpatrick KL (2007). An examination of self-compassion in relation to positive psychological functioning and personality traits. J Res Pers.

[64] Zessin U, Dickhäuser O, Garbade S (2015). The relationship between self-compassion and well-being: a meta-analysis. Appl Psychol Health Well Being.

[65] Crane RS, Brewer J, Feldman C (2017). What defines mindfulness-based programs? The warp and the weft. Psychol Med.

[66] Linardon J (2023). Rates of attrition and engagement in randomized controlled trials of mindfulness apps: systematic review and meta-analysis. Behav Res Ther.

[67] Daros AR, Patel A, Otevwe O, Sotelo S, Saab BJ, Quilty LC (2025). Acceptability, engagement, outcomes, and dose–response associations of a mindfulness-based meditation app in individuals waiting for psychological services. BMC Digit Health.

[68] Osborne LA, McHugh L, Saunders J, Reed P (2008). Parenting stress reduces the effectiveness of early teaching interventions for autistic spectrum disorders. J Autism Dev Disord.

[69] McClain MB, Harris B, Schwartz SE, Benallie KJ, Golson ME, Benney CM (2019). Brief report: development and validation of the Autism Spectrum Knowledge Scale General Population Version: preliminary analyses. J Autism Dev Disord.

[70] Kirkman JJL, Dadds MR, Hawes DJ (2018). Development and validation of the knowledge of parenting strategies scale: measuring effective parenting strategies. J Child Fam Stud.

[71] Kurzrok J, McBride E, Grossman RB (2021). Autism-specific parenting self-efficacy: an examination of the role of parent-reported intervention involvement, satisfaction with intervention-related training, and caregiver burden. Autism.

[72] Cannon HM, Feinstein AH, Friesen DP (2010). Managing complexity: applying the conscious-competence model to experiential learning. DBSEL.

[73] Glasgow RE, Harden SM, Gaglio B (2019). RE-AIM planning and evaluation framework: adapting to new science and practice with a 20-year review. Front Public Health.

[74] Curran GM, Bauer M, Mittman B, Pyne JM, Stetler C (2012). Effectiveness-implementation hybrid designs: combining elements of clinical effectiveness and implementation research to enhance public health impact. Med Care.

[75] Moore RC, Depp CA, Wetherell JL, Lenze EJ (2016). Ecological momentary assessment versus standard assessment instruments for measuring mindfulness, depressed mood, and anxiety among older adults. J Psychiatr Res.

