# Supplementary material for: Mindfulness-Based Psychoeducation App to Improve the Well-Being of Parents and Caregivers of Children With Autism: Development and Usability Study
Source: JMIR Pediatr Parent. 2026 Jun 4;9:e84224. doi: 10.2196/84224 (PMC13235980; doi:10.2196/84224)
Supplement: Multimedia Appendix 1 [file pediatrics-v9-e84224-s001.docx]

**Multimedia Appendix 1.**

Qualitative study topic guide for semi-structured interviews.

| Topic | Questions |
| --- | --- |
| Diagnosis | - What was it like for parents when they first got informed about their child’s ASD diagnosis? How do they react and/or feel? - What are parents’ own perception of their child’s diagnosis? |
| Symptom management | - What are parents’ main difficulties in caregiving with regards to their child’s ASD symptoms? Why is that the case? - How do they cope/respond to these situations? - What do parents need the most in order to overcome these difficulties? |
| Family dynamics | - How does the wider family dynamics affect parents’ experience of caregiving for ASD? - Are there any factors or situations that are particularly helpful or causing extra stress? |
| Stigma | - What are parents’ experiences of stigma related to ASD? - What are their thoughts and feelings when faced with public stigma? |
