# Supplementary material for: Mindfulness-Based Psychoeducation App to Improve the Well-Being of Parents and Caregivers of Children With Autism: Development and Usability Study
Source: JMIR Pediatr Parent. 2026 Jun 4;9:e84224. doi: 10.2196/84224 (PMC13235980; doi:10.2196/84224)
Supplement: Multimedia Appendix 2 [file pediatrics-v9-e84224-s002.docx]

**Multimedia Appendix 2.**

Qualitative study codes and examples mapped to an adapted socioecological model (SEM).

| SEM level | Codes | Examples |
| --- | --- | --- |
| **Individual** |  |  |
| Knowledge | Lack of knowledge about ASD | Sensory issues; local and global processing |
|  | Misunderstanding about ASD | Social interactions; repetitive behaviours; individual differences within ASD individuals |
| Skills | ***Challenges in terms of skills for managing child behaviour*** | |
|  | Lack of parenting skills | Communication skills training, organising play for children; toilet training; behavioural issues |
|  | Misunderstanding about management of behaviours and symptoms | Misconstrued behavioural reinforcement; ineffective instructions and commands; emotionally-charged parenting; negative parenting practices; repetitive behaviours and restricted interests; social interactions; language and communication |
|  | ***Need for skills during daily interaction with children*** | |
|  | Boundaries and limit setting | Consequences of not setting boundaries; how to set and maintain effective boundaries |
|  | Perspective taking | Adopting an investigative approach; double empathy problem; importance of perspective taking |
|  | Strengths-based parenting | Strengths of ASD; importance of seeing children’s strengths; context of the Chinese culture |
|  | ***Need for skills to take care of parents themselves*** | |
|  | Expectation management | Why parents should manage expectations; what parents can expect with ASD |
|  | Self-care | Importance of self-care; suggestions for methods of how parents can take care of themselves |
| Emotions | Negative emotions | Lack of support for ASD, child’s development and progress; lack of validation of parenting efforts; complicated emotions arising from stigma |
|  | Lack of emotional awareness | Importance of emotional awareness; how emotional awareness can help parents be mindful of their actions |
| Attitudes | Lack of acceptance | Resistance and reluctance to acknowledge ASD; process of acceptance and how therapy can be helpful |
|  | Lack of curiosity | Need for adopting an investigate mindset; observe children’s both strengths and weaknesses |
| **Interpersonal** | Inconsistent parenting | Parents disregarding each other; imbalanced familial roles as parents taking up different roles in caregiving; different caregiving approach from grandparents; need for both parents to show love and set limits |
|  | Autism in the family | Unhelpful perception of ASD by fathers who have ASD; impact of ASD on neurotypical siblings in the family |
| **Institutional** | Service gap | Long waiting time between triage and first appointment with minimal support; providing practical tips and specific skills can be helpful |
|  | Transition to primary school | Changes in accommodation for children’s behaviours in preschool vs primary school; |
| **Community** | Support system | Communication, openness, and support within the couple; peer support groups with other parents who face similar challenges; practical support from healthcare professionals |
|  | Cultural influences | Tendency to express emotions indirectly in Chinese culture (especially when giving compliments) |
| **Societal** | Stigma | Negative comments and labels for ASD; negative impact of labelling ASD as a mental illness |
