# Supplementary material for: Mindfulness-Based Psychoeducation App to Improve the Well-Being of Parents and Caregivers of Children With Autism: Development and Usability Study
Source: JMIR Pediatr Parent. 2026 Jun 4;9:e84224. doi: 10.2196/84224 (PMC13235980; doi:10.2196/84224)
Supplement: Multimedia Appendix 3 [file pediatrics-v9-e84224-s003.docx]

**Multimedia Appendix 3.**

Practical application of social cognitive theory (SCT) and behaviour change techniques (BCT) to achieve change objectives in the TRIP app.

| SCT | BCT | Practical applications to achieve change objectives in TRIP app [corresponding to change objectives in Table 1] |
| --- | --- | --- |
| Behavioural capability | Instruction on how to perform a behaviour [4.1] | - Knowledge components fill in gaps in knowledge about ASD [1.1.1 and 1.1.3]. - Skills components focus on how to use effective instructions and commands when parenting [1.2.1]. |
|  | Information about antecedents [4.2] | - Knowledge components explain any misunderstanding about ASD [1.1.2]. - Skills components explain any mismanagement of behaviours and specific ASD symptoms [1.2.2 and 1.2.3]. |
|  | Behavioural substitution [8.2], habit reversal [8.4], and reduce negative emotions [11.2] | - Mindfulness components provide an alternative to living on autopilot and being driven by automatic reactivity, substituting negative parenting practices driven by emotions [2.2.1 and 2.3.1]. - Mindfulness components also provide an alternative to exhaustion and burnout through advocating self-care, self-compassion, and appropriate limit-setting [2.2.3, 2.2.4, 2.3.2]. |
| Outcome expectations | Information about  health [5.1], social [5.3], and emotional [5.6] consequences | - Knowledge and skills components explain the problems and ineffectiveness of unhelpful and negative parenting practices and inconsistent parenting [2.1.1, 2.1.2, and 2.2.1]. - Knowledge and skills components explain ASD prognosis and appropriate expectations for child’s progress and development [2.2.2]. |
|  | Comparative imagining of future outcomes [9.3] | - Mindfulness exercise include imagination exercise that prompt parents in imagining how things can be different as they embody mindfulness. |

| SCT | BCT | Practical applications to achieve change objectives in TRIP app [corresponding to change objectives in Table 1] |
| --- | --- | --- |
| Observational learning | Demonstration of the behaviour [6.1] | - Skills and relational skills components include case examples of parenting strategies by healthcare professionals [1.2.1, 1.2.2, 1.2.3, and 2.2.1]. |
|  | Salience of consequences [5.2], and vicarious reinforcement [16.3] | - Skills and relational skills components include case examples of unskilful parenting shared by healthcare professionals to highlight how such parenting is perceived by, and may affect, children [1.2.1, 1.2.2, 1.2.3, 2.2.1, and 2.2.2]. |
| Self-efficacy | Reattribution [4.3] | - Mindfulness components help parents understand that stress and conflicts in parenting can be driven by automatic reactivity to negative emotions [2.2.1] and can be reattributed to parents’ emotions and attitudes [1.4.1, 2.3.1, 2.4.1 and 2.4.2]. |
|  | Problem solving [1.2] | - Mindfulness exercises prompt parents to analyse their own parenting behaviours in stressful parenting scenarios in everyday life and recognise factors contributing to parental stress and emotions [2.2.2 and 2.3.1]. |
|  | Behavioural practice/ rehearsal [8.1] and habit formation [8.3] | - Mindfulness practices and exercises repeated within the core curriculum to build the skills of attentional control, decentring, and self-compassion to support parents in cultivating the qualities of mindfulness which can in turn improve parenting for ASD [1.4.1, 2.2.1, 2.2.2, 2.2.3, 2.3.1, 2.3.2, 2.4.2]. - Dedicated section in the app to facilitate repeated practice through allowing easy and convenient revisiting of mindfulness practices. |
| Reinforcements | Action planning [1.4] and habit formation [8.3] | - Reminder function allows practices to be planned for completion at a set time of each day to incorporate mindfulness practice in daily life. |
|  | Prompts/cues [7.1] | - Daily push notifications sent at set time each day as a cue to remind parents to login and complete sessions or practices. |
|  | Social reward [10.4]  and rewarding completion [14.5] | - Congratulatory message and gamified collectibles delivered after a session is completed as an incentive for maintaining extrinsic motivation. |
