# Supplementary material for: Mindfulness-Based Psychoeducation App to Improve the Well-Being of Parents and Caregivers of Children With Autism: Development and Usability Study
Source: JMIR Pediatr Parent. 2026 Jun 4;9:e84224. doi: 10.2196/84224 (PMC13235980; doi:10.2196/84224)
Supplement: Multimedia Appendix 4 [file pediatrics-v9-e84224-s004.docx]

**Multimedia Appendix 4.**

Pilot waitlist-randomised controlled trial recruitment flow, baseline characteristics of participants and their children, and results in terms of feasibility, acceptability, and preliminary efficacy.

a) CONSORT flow diagram of the pilot study.


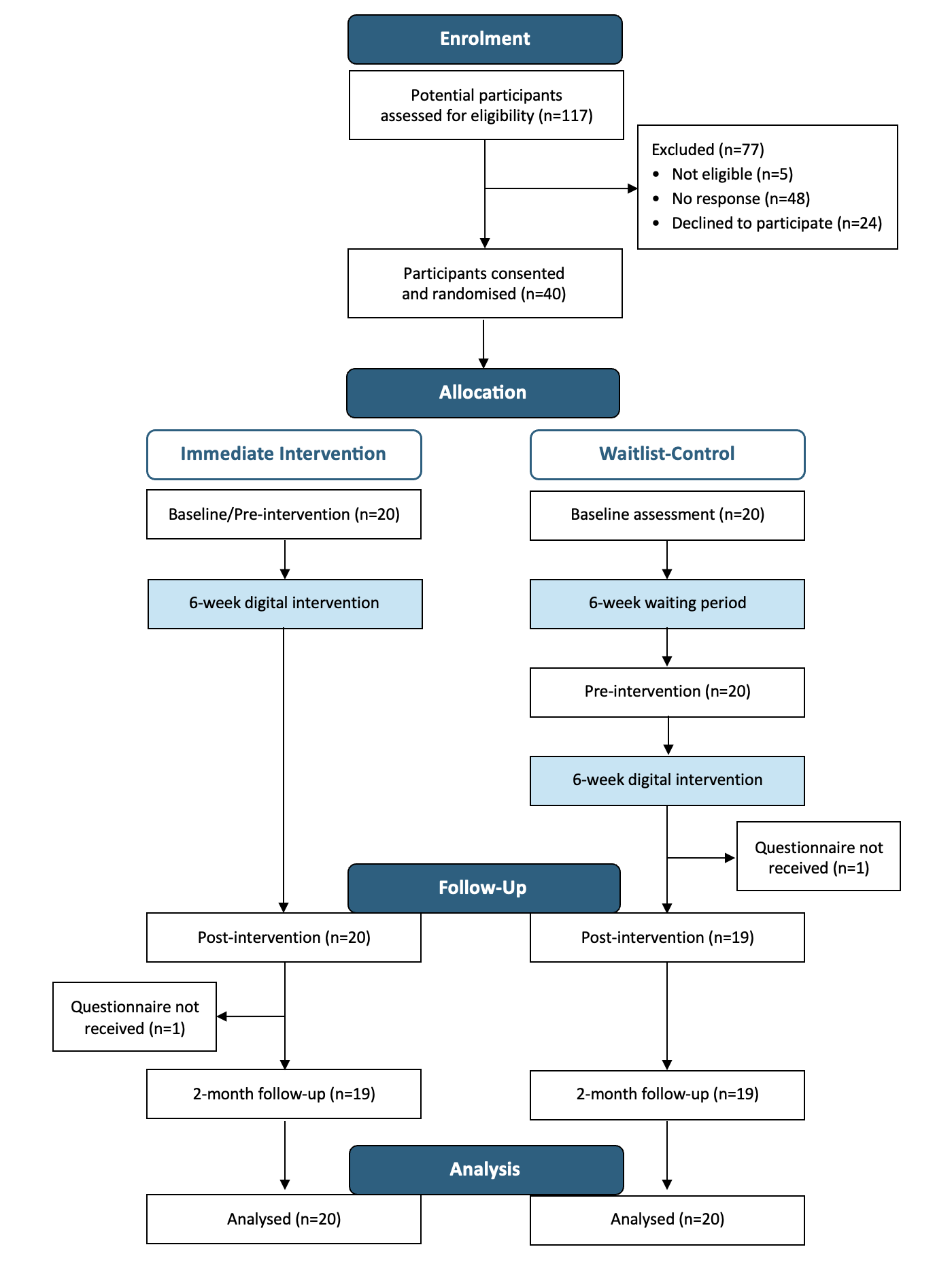


b) Individual participant characteristics at baseline (n = 40).

| Participant characteristics | Waiting list (n = 20) | | Clinical care (n = 20) | |
| --- | --- | --- | --- | --- |
|  | **Intervention**  **(n = 10)** | **Control**  **(n = 10)** | **Intervention**  **(n = 10)** | **Control**  **(n = 10)** |
| Parent characteristics |  |  |  |  |
| Gender, n(%) |  |  |  |  |
| Male | 2 (20%) | 0 (0%) | 1 (10%) | 1 (10%) |
| Female | 8 (80%) | 10 (100%) | 9 (90%) | 9 (90%) |
| Age, n(%) |  |  |  |  |
| 21-30 years | 0 (0%) | 1 (10%) | 0 (0%) | 0 (0%) |
| 31-40 years | 8 (80%) | 8 (80%) | 5 (50%) | 2 (20%) |
| 41-50 years | 2 (20%) | 1 (10%) | 5 (50%) | 7 (70%) |
| 51-60 years | 0 (0%) | 0 (0%) | 0 (0%) | 1 (10%) |
| Education level, n(%) |  |  |  |  |
| Secondary | 3 (30%) | 5 (50%) | 3 (30%) | 4 (40%) |
| Post-secondary | 7 (70%) | 5 (50%) | 7 (70%) | 6 (60%) |
| Characteristics of child with ASD |  |  |  |  |
| Gender, n(%) |  |  |  |  |
| Male | 5 (50%) | 8 (80%) | 10 (100%) | 7 (70%) |
| Female | 5 (50%) | 2 (20%) | 0 (0%) | 3 (30%) |
| Age, mean (SD) | 3.90 (1.85) | 3.00 (1.05) | 6.90 (1.79) | 6.90 (2.18) |
| Clinical characteristics, mean (SD) |  |  |  |  |
| ASD core symptoms (SRS-2) | 77.90 (17.14) | 86.50 (37.45) | 84.40 (31.06) | 83.30 (23.55) |
| Externalising disorders (CBCL-Ext) | 16.20 (11.50) | 18.00 (17.00) | 16.70 (12.60) | 9.80 (6.71) |
| Attention problems (CBCL-Attn) | 4.90 (3.18) | 4.50 (2.64) | 9.00 (6.75) | 7.20 (3.12) |
| Anxiety (ASC-ASD) | 14.00 (5.68) | 15.70 (6.33) | 22.60 (12.04) | 17.60 (10.49) |

Note: all ps > .05 for all comparisons between intervention and control with chi-square tests for categorical variable and t-tests for continuous variables (stratified by the levels of support children are currently receiving).

c) Feasibility measures in the active intervention and follow-up period respectively (n = 40).

| Feasibility outcomes (n = 40) | 6-week intervention period | | 2-month follow-up period | |
| --- | --- | --- | --- | --- |
| **Usage statistics** | **N** | **%** | **N** | **%** |
| Day of week of use |  |  |  |  |
| Weekdays | 531 | 78.3 | 39 | 72.2 |
| Weekend | 147 | 21.7 | 15 | 27.8 |
| Time of day of use |  |  |  |  |
| 06:00-10:00 | 164 | 24.2 | 16 | 29.6 |
| 10:00-14:00 | 90 | 13.3 | 6 | 11.1 |
| 14:00-18:00 | 149 | 22.0 | 8 | 14.8 |
| 18:00-22:00 | 6 | 0.9 | 2 | 3.7 |
| 22:00-02:00 | 138 | 20.4 | 12 | 22.2 |
| 02:00-06:00 | 131 | 19.3 | 10 | 18.5 |
|  | **Mean** | **SD** | **Mean** | **SD** |
| **User engagement** |  |  |  |  |
| Number of usage days | 14.0 | 7.01 | 1.2 | 2.08 |
| Total number of logins | 17.4 | 8.26 | 1.4 | 2.71 |
| Total time spent (mins) | 165.9 | 58.00 | 8.9 | 27.83 |
| **Program participation** |  |  |  |  |
| Unique components completed | 65.3 | 11.51 | - | - |
| Core sessions completed | 16.9 | 2.48 | - | - |
| Optional sessions completed | 2.4 | 2.60 | - | - |
| **User preferences** |  |  |  |  |
| Components by presentation format |  |  |  |  |
| Audio | - | - | 1.36 | 4.02 |
| Video | - | - | 0.21 | 0.86 |
| Text | - | - | 2.36 | 5.65 |
| Components by content type |  |  |  |  |
| Knowledge | - | - | 0.15 | 0.59 |
| Skills | - | - | 1.77 | 3.73 |
| Emotions | - | - | 0.03 | 0.16 |
| Attitudes | - | - | 0.44 | 1.33 |
| Mindfulness Practice | - | - | 1.00 | 3.09 |
| Mindfulness Debrief | - | - | 0.03 | 0.16 |
| Mindfulness Exercises | - | - | 0.21 | 0.77 |
| Mindful relational skills | - | - | 0.31 | 0.95 |

c) Acceptability measured by uMARS item ratings for each of the four subdomains: Engagement, Functionality, Aesthetics, Information (top), and subjective app quality (bottom) (n = 39).


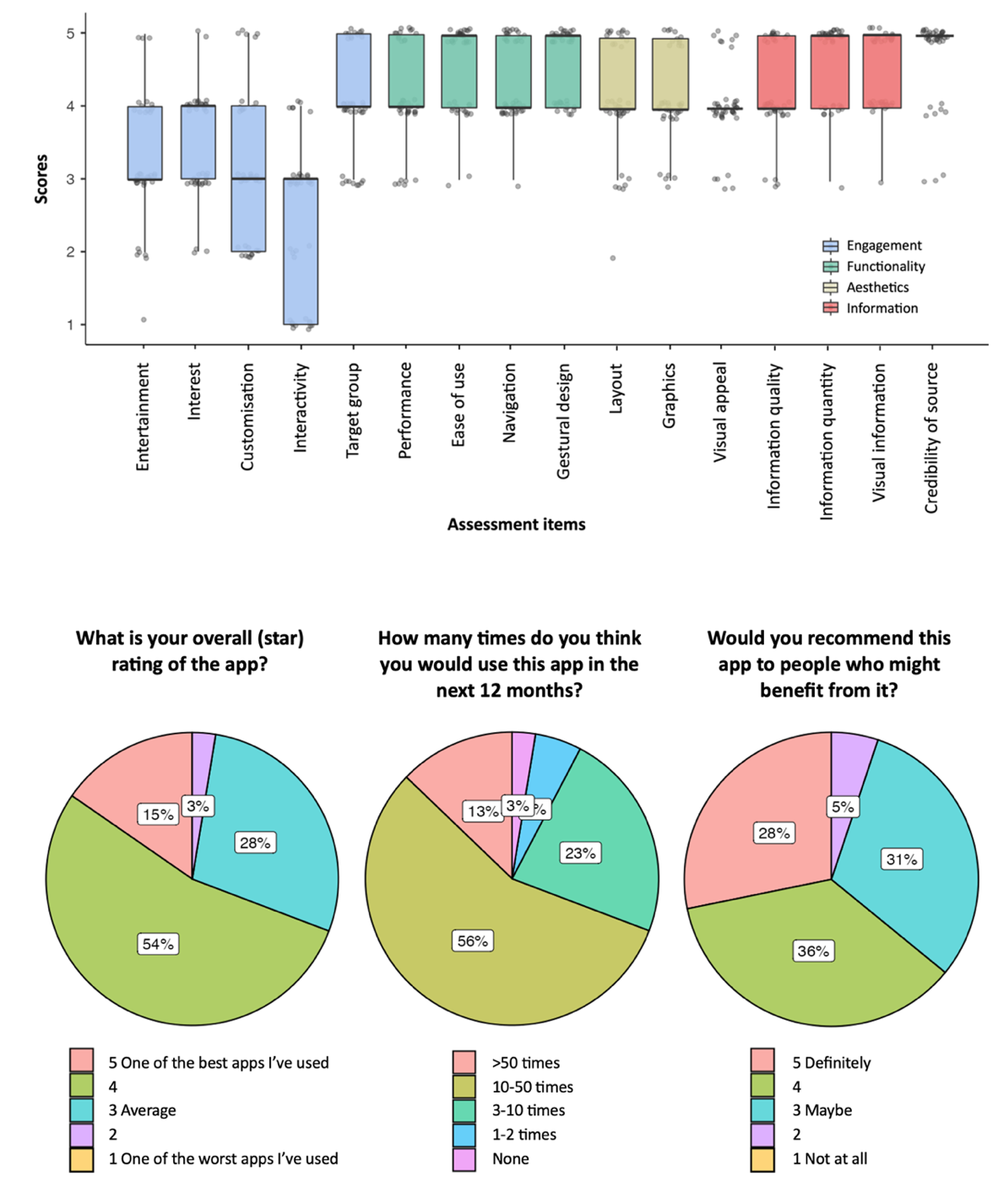


d) Intervention effect stratified by the levels of support children are currently receiving (n = 40).

| Outcomes | | Waiting list (n = 20) | | Adjusted  int. effect | Clinical care (n = 20) | | Adjusted  int. effect |
| --- | --- | --- | --- | --- | --- | --- | --- |
|  |  | **Int** | **Control** |  | **Int** | **Control** |  |
|  |  | M (SD) | M (SD) | B (95% CI) | M (SD) | M (SD) | B (95% CI) |
| Parental mental health | | | | | | | |
| GAD-7 | Baseline | 6.80 (5.07) | 6.80 (6.99) | -0.95  (-3.62, 1.73) | 6.50 (5.76) | 5.50 (3.60) | 0.67  (-2.88, 4.23) |
|  | 6-week | 6.30 (2.83) | 7.00 (2.40) |  | 5.30 (2.58) | 4.60 (4.81) |  |
| PHQ-9 | Baseline | 5.00 (3.20) | 7.90 (6.72) | -1.30  (-3.83, 1.23) | 6.40 (5.83) | 5.30 (5.68) | -0.19  (-4.65, 4.27) |
|  | 6-week | 4.00 (2.05) | 5.60 (2.80) |  | 5.20 (4.39) | 5.10 (4.84) |  |
| PSI-SF | Baseline | 101.40 (22.91) | 97.20 (31.80) | -1.60  (-16.61, 13.40) | 107.90 (26.63) | 98.70 (22.44) | -1.28  (-20.60, 18.05) |
|  | 6-week | 105.50 (16.77) | 103.60 (25.09) |  | 105.40 (20.48) | 96.60 (24.44) |  |
| Parenting capabilities | | | | | | | |
| PSOC | Baseline | 53.60 (9.72) | 57.10 (10.70) | 0.91  (-9.43, 11.24) | 56.70 (7.99) | 56.60 (7.37) | 2.55  (-7.07, 12.17) |
|  | 6-week | 54.50 (12.19) | 55.30 (7.27) |  | 56.00 (8.64) | 54.30 (8.38) |  |
| PSA-PE | Baseline | 27.20 (5.14) | 30.20 (5.55) | -1.17  (-4.83, 2.49) | 30.20 (3.94) | 30.30 (4.90) | 0.66  (-2.33, 3.66) |
|  | 6-week | 28.50 (4.06) | 30.80 (4.24) |  | 31.30 (1.83) | 30.60 (3.81) |  |
| PSDQ-W | Baseline | 27.20 (4.54) | 31.70 (3.86) | 0.34  (-2.35, 3.04) | 28.30 (4.83) | 26.50 (4.43) | 3.17*  (1.04, 5.30) |
|  | 6-week | 28.20 (4.26) | 31.60 (3.69) |  | 29.30 (3.13) | 25.70 (2.83) |  |
| Mindfulness levels | | | | | | | |
| MAAS | Baseline | 3.03 (0.89) | 3.09 (0.75) | 0.09  (-0.51, 0.69) | 3.00 (0.88) | 2.75 (0.99) | 0.02  (-0.53, 0.58) |
|  | 6-week | 3.38 (0.57) | 3.29 (0.95) |  | 3.13 (0.77) | 2.95 (0.90) |  |
| IM-P | Baseline | 87.50 (7.25) | 97.60 (10.56) | -11.62*  (-19.34, -3.90) | 90.80 (11.76) | 89.40 (10.45) | -12.63*  (-19.13, -6.13) |
|  | 6-week | 89.60  (8.73) | 107.20 (8.66) |  | 91.80 (11.97) | 101.70 (10.60) |  |

Note: Int = intervention; 6-week = 6-week post-baseline; outcomes measured are as follows: GAD-7 – anxiety symptoms; PHQ-9 – depressive symptoms; PSI-SF – parenting stress; PSOC – sense of competence; PSA-PE – parenting efficacy; PSDQ-W – parenting warmth; MAAS – mindfulness; IM-P – mindfulness in parenting; intervention effects were adjusted for baseline value of the outcome, as well as child’s ASD core symptoms (SRS-2) and externalising behaviours (CBCL-Ext) at baseline; *p < 0.05.

e) Outcome changes over time from pre-intervention stratified by the levels of support children are currently receiving (n = 40).

| Changes in outcomes  from pre-intervention | | Waiting list  (n = 20) | Adjusted change over time | Clinical care  (n = 20) | Adjusted change over time |
| --- | --- | --- | --- | --- | --- |
|  |  | M (SD) | B (95% CI) | M (SD) | B (95% CI) |
| Parental mental health | |  |  |  |  |
| GAD-7 | Post-intervention | -0.60 (4.97) | -1.80*  (-3.23, -0.37) | -0.85 (3.25) | -0.35  (-2.30, 1.60) |
|  | 2-month | -2.40 (4.35) |  | -1.20 (4.30) |  |
| PHQ-9 | Post-intervention | -0.20 (4.63) | -1.10  (-2.90, 0.70) | -1.35 (3.54) | 1.00  (-1.68, 3.68) |
|  | 2-month | -1.30 (3.70) |  | -0.35 (5.37) |  |
| PSI-SF | Post-intervention | -1.55 (18.27) | -6.85  (-16.45, 2.75) | -6.50 (14.44) | -1.65  (-9.77, 6.47) |
|  | 2-month | -8.40 (14.67) |  | -8.15 (12.97) |  |
| Parenting capabilities | |  |  |  |  |
| PSOC | Post-intervention | 2.20 (14.45) | 0.75  (-6.43, 7.93) | 1.80 (10.83) | 0.40  (-3.83, 4.63) |
|  | 2-month | 2.95 (12.78) |  | 2.20 (12.80) |  |
| PSA-PE | Post-intervention | 0.30 (3.39) | 0.65  (-1.18, 2.48) | 0.95 (3.53) | -1.00  (-2.67, 0.67) |
|  | 2-month | 0.95 (3.17) |  | -0.05 (2.82) |  |
| PSDQ-W | Post-intervention | 0.25 (3.58) | -1.10  (-2.93, 0.73) | 1.25 (2.81) | -0.30  (-1.89, 1.29) |
|  | 2-month | -0.85 (3.12) |  | 0.95 (2.76) |  |
| Mindfulness levels | |  |  |  |  |
| MAAS | Post-intervention | 0.10 (0.77) | -0.11  (-0.54, 0.32) | 0.08 (0.53) | -0.04  (-0.34, 0.26) |
|  | 2-month | -0.01 (0.65) |  | 0.05 (0.44) |  |
| IM-P | Post-intervention | -6.45 (12.53) | 2.75  (-1.85, 7.35) | -6.10 (10.16) | 0.05  (-4.44, 4.54) |
|  | 2-month | -3.70 (11.47) |  | -6.05 (9.66) |  |

Note: 2-month = 2-month post-intervention; outcomes measured are as follows: GAD-7 – anxiety symptoms; PHQ-9 – depressive symptoms; PSI-SF – parenting stress; PSOC – sense of competence; PSA-PE – parenting efficacy; PSDQ-W – parenting warmth; MAAS – mindfulness; IM-P – mindfulness in parenting; change over time from pre-intervention adjusted for pre-intervention value of the outcome, child’s ASD core symptoms (SRS-2) and externalising behaviours (CBCL-Ext) at baseline, and randomisation group (intervention vs control); *p < 0.05.
