# Supplementary material for: Mindfulness-Based Psychoeducation App to Improve the Well-Being of Parents and Caregivers of Children With Autism: Development and Usability Study
Source: JMIR Pediatr Parent. 2026 Jun 4;9:e84224. doi: 10.2196/84224 (PMC13235980; doi:10.2196/84224)
Supplement: Multimedia Appendix 5 [file pediatrics-v9-e84224-s005.docx]

**Multimedia Appendix 5.**

Implementation plan for different settings.

The implementation plan was devised according to the Reach, Effectiveness, Adoption, Implementation, and Maintenance (RE-AIM) framework (Glasgow et al., 2019).

| RE-AIM Framework | Clinical settings | | Community settings | |
| --- | --- | --- | --- | --- |
|  | **Clinic** | **Triage** | **Non-profit** | **Community centres** |
| Implementers | Clinicians, including psychiatrists, nurses, and allied health professionals | | Staffs of charities, non-governmental organisations, and local community centres | |
| Reach | Families who attended appointments with psychiatric services but may not be receiving adequate support due to limited resources. | Families of children suspected of ASD who are on the waiting list for diagnostic assessment. | Families who are served by charities and other non-governmental organisations providing services for the ASD population. | Families who may identify their children as neurodiverse but are unbeknownst to clinical services or  non-profit. |
| Effectiveness | Mental health outcomes will be measured by a brief survey that will be incorporated in the app at the start and the end of the structured intervention. | | | |
|  | Further outcomes will be measured by additional surveys distributed at registration when families arrive at the hospital for appointments. | | Further outcomes will be measured by additional surveys distributed at the reception and at events hosted by the non-profit and community centres. | |
| Adoption | Site visits and service mapping will be conducted prior to implementation. App demonstration videos and implementation manuals will include data about the efficacy of the app, and templates for how to introduce the aims and objectives of the app. | | | |
|  | Implementation manuals will be tailored for clinicians to include a detail curriculum of the app, including psychological theories and specific parenting skills covered, as well as suggestions on how they can incorporate the app during their routine clinical service provision. | | Implementation manuals will be tailored for staffs at non-profit and community centres to include a brief topic guide of content covered in the app, answers to frequently asked questions, and a detailed guide for app installation and setup. | |
| Implementation | Promotional resources (e.g. flyers and booklets) will be provided to implementers in different settings. Implementers will also be able to access ongoing technical support from the research team, and to provide feedback with regards to the implementation process and any intervention content to be added. | | | |
|  | Clinicians will be asked to document in clinical case notes when they refer families to the app, so that the implementation can be evaluated. | | Staffs at non-profit and community centres will be asked to keep a log of the number of parents they introduced the app to. | |
| Maintenance | In-app notifications will encourage parents to revisit content and practice mindfulness daily. The research team will also develop new content based on feedback received during implementation, parents will be informed of new content through in-app notifications. | | | |
|  | Clinical and community champions will encourage parents to form support groups in their respective settings. The research team will also collaborate with implementers to host in-person mindfulness workshops to promote ongoing use of app. | | | |
